# Investigation of Chiral Smectic Phases and Conformationally Disordered Crystal Phases of the Liquid Crystalline 3F5FPhH6 Compound Partially Fluorinated at the Terminal Chain and Rigid Core

**DOI:** 10.1021/acs.jpcb.2c03654

**Published:** 2022-08-19

**Authors:** Aleksandra Deptuch, Małgorzata Jasiurkowska-Delaporte, Ewa Juszyńska-Gałązka, Anna Drzewicz, Marcin Piwowarczyk, Magdalena Urbańska, Stanisław Baran

**Affiliations:** † Institute of Nuclear Physics Polish Academy of Sciences, PL-31342 Kraków, Poland; ‡ Research Center for Thermal and Entropic Science, Graduate School of Science, Osaka University, 560-0043 Osaka, Japan; § Institute of Chemistry, Military University of Technology, PL-00908 Warsaw, Poland; ∥ Marian Smoluchowski Institute of Physics, Jagiellonian University, PL-30348 Kraków, Poland

## Abstract

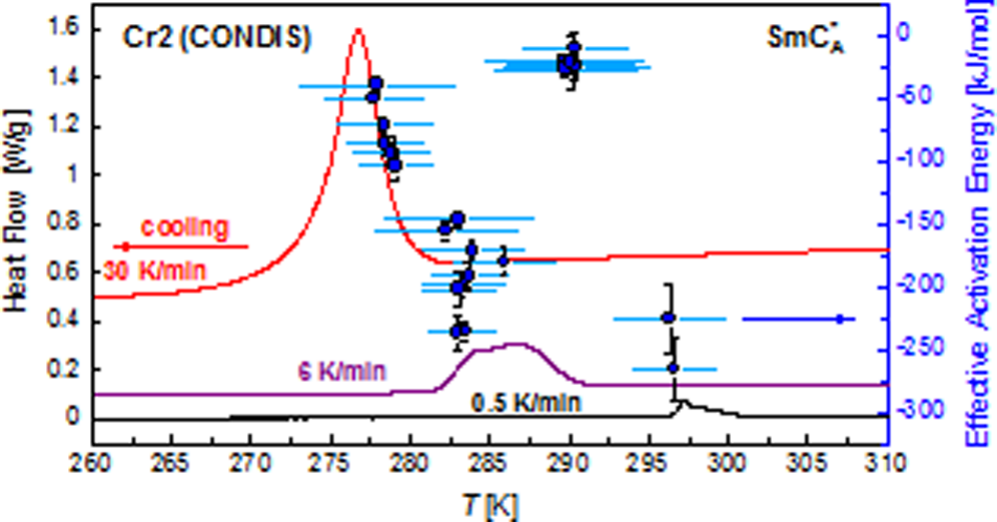

Complementary methods are applied to investigate the phase transitions and crystallization kinetics of the liquid crystalline compound denoted as 3F5FPhH6. Two crystal phases are confirmed, and one of them is the conformationally disordered (CONDIS) phase. Complexity of the melt crystallization process is revealed by the analysis with Friedman’s isoconversional method. The melt crystallization of 3F5FPhH6 shows different mechanisms depending on temperature, which is explained by the relation between the thermodynamic driving force and the thermal energy of translational degrees of freedom. The studied compound crystallizes even during fast cooling (30 K/min), unlike similar compounds with different fluorosubstitutions of the benzene ring, which form the smectic glass for moderate cooling rates. The tendency to vitrification of the smectic phase decreases apparently with the decreasing stability width of the SmC_A_* phase and the increasing relaxation time of the collective relaxation process in this phase, at least for homologues differing from 3F5FPhH6 only by the type of fluorosubstitution.

## 1 Introduction

**Figure 1:**
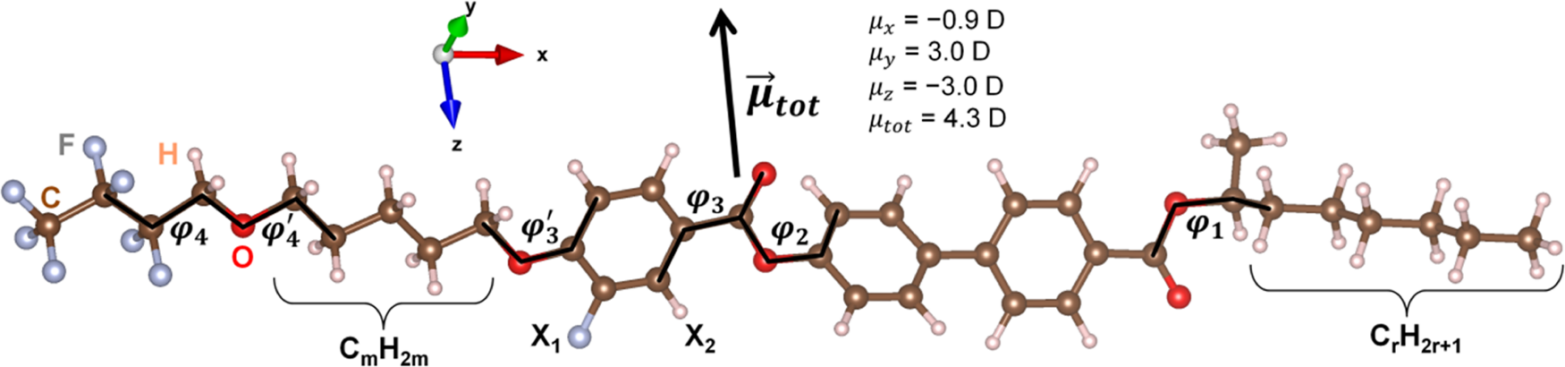
Isolated 3F5FPhH6 molecule optimized with the DFT-B3LYP/TZVPP method. The total dipole moment vector is indicated. The 3F*m*X_1_PhX_2_*r* compounds differ by the length of the −C_m_H_2m_– and −C_r_H_2r+1_ chains as well as by fluorosubstitution at the X_1_ and X_2_ positions.

Determination of correlation between the molecular structure of liquid crystals and their properties is not straightforward, as their molecules consist of numerous atoms and at the same time the exchange of even one atom may lead to significant differences in the compounds’ behavior. An example of that is the chiral liquid crystalline compound abbreviated as 3F*m*X_1_PhX_2_*r*, characterized by the molecular core consisting of the benzene ring and biphenyl connected by a −COO– bridge and two terminal chains, with a partially fluorinated nonchiral chain (Figure 1).^1−3^ Such a molecular structure leads often to the presence of the antiferroelectric smectic C_A_* (SmC_A_*) phase in a broad temperature range and with a high tilt angle;^4−6^ therefore, the main purpose of the synthesis of the 3F*m*X_1_PhX_2_*r* compounds was their future potential use in the display technology, where the orthoconic SmC_A_* phase (characterized by a tilt angle of ca. 45°) enables the perfect dark state in a display.^7,8^ The 3F*m*X_1_PhX_2_*r* compounds with *m* = 5 studied in details are 3F5HPhF4,^9^ 3F5HPhF6,^10^ 3F5FPhF6,^11^ 3F5HPhH6,^12^ and 3F5HPhH7.^13,14^ Three of them are confirmed glassformers: 3F5HPhF4 and 3F5HPhF6 exhibit the vitrified SmC_A_* phase^9,10^ and 3F5HPhH6 and 3F5HPhH7 exhibit the vitrified hexatic SmX_A_* phase, which is either SmI_A_* or SmF_A_*.^12−14^ For 3F5FPhF6, the vitrification of the SmC_A_* phase has not been shown explicitly yet, but it is implied by a very low crystallization rate on cooling.^11^ The results from ref^10−12^ show that the 3F*m*X_1_PhX_2_*r* compounds with *m* = 5 and *r* = 6 have a tendency to the glass transition of the chiral smectic phase for various fluorosubstitutions of the benzene ring, either both at the X_1_, X_2_ positions, only at the X_2_ position, or with no F atoms in the benzene ring.

This study involves the last 3F*m*X_1_PhX_2_*r* compound with *m* = 5 and *r* = 6, which is 3F5FPhH6, fluorosubstituted only at the X_1_ position (Figure 1). The phase sequence of 3F5FPhH6, determined at 1 K/min rate, is Cr (336.8 K) SmC_A_* (382.0 K) SmC* (382.1 K) SmA* (384.6 K) Iso.^2^ The investigation of 3F5FPhH6 presented in this paper involves differential scanning calorimetry (DSC), polarizing optical microscopy (POM), X-ray diffraction (XRD), and broad-band dielectric spectroscopy (BDS). Our aim is to check whether 3F5FPhH6 undergoes the glass transition in the smectic phase, as its counterparts with different types of fluorination,^10−12^ to study the kinetics of crystallization and polymorphism in the solid state and to investigate the dielectric relaxation processes in the smectic and crystal phases. The results are further compared with other 3F*m*X_1_PhX_2_*r* compounds with *m* = 5.^9−14^

## 2 Experimental Details

(*S*)-4′-(1-Methylheptylcarbonyl)biphenyl-4-yl 4-[5-(2,2,3,3,4,4,4-heptafluorobutoxy)pentyl-1-oxy]-3-fluorobenzoate, abbreviated as 3F5FPhH6, was synthesized according to the route described in ref.^1,2^ The purity of the compound (99.9%) was tested by the mass spectrometry, infrared spectroscopy, and proton magnetic resonance methods, as described in detail in the electronic Supporting Information of ref (2).

DSC curves were collected with the DSC 2500 TA Instrument calorimeter for the 5.04 mg sample of 3F5FPhH6, contained within an aluminum pan. DSC measurements were performed in the 153–403 or 153–413 K range with 2, 5, 10, 15, and 20 K/min rates, first during cooling and subsequently during heating for each rate, after primary heating the sample to the isotropic liquid. For investigation of crystallization in nonisothermal conditions and of transitions between the crystal phases, additional DSC measurements were performed in the 228–363 K range during cooling and heating at 0.5–30 K/min. Finally, the crystallization kinetics in isothermal conditions was investigated: the sample was heated above the melting temperature and cooled at 20 K/min to a selected crystallization temperature *T*_cr_ from the 285–295 K range. After the crystallization in a given *T*_cr_ was completed, the sample was heated up at 20 K/min above the melting temperature. The DSC results were analyzed with TRIOS software.

POM observations were performed with a PZO Mikroskopy polarizing microscope during cooling and heating at the 20 K/min rate. Numerical analysis of POM textures was performed with “rgb” and “gray” algorithms of TOApy^15^ and with the fractal box count method^16^ in ImageJ.^17^

XRD measurements were performed using the Cu Kα radiation with an Empyrean 2 (PANalytical) diffractometer with the Cryostream 700 Plus (Oxford Cryosystems) temperature attachment. The sample in the isotropic phase was introduced to the capillary (borosilicate glass, 0.3 mm diameter) by the capillary effect, and afterward the sample was left at room temperature for about a week. Then, the diffraction patterns in the 2θ = 2–8 or 2–30° range were collected in selected temperatures from the 170–400 K range. XRD data were analyzed with WinPLOTR.^18^

BDS spectra in the 1–10^7^ Hz frequency range were collected with the Novocontrol Technologies spectrometer for 50 μm thick samples between gold electrodes. The measurements were performed on cooling and subsequent heating. Additionally, the spectra in and near the temperature range of the SmC* phase were collected on cooling in the bias field of 0.8 V/μm to observe the temperature dependence of the soft mode with the stronger Goldstone mode suppressed.

Models of the isolated molecule of 3F5FPhH6 were prepared in Avogadro^19^ and optimized with the semiempirical PM7 method in MOPAC2016.^20^ Next, for the conformations in the local minima of potential energy, the density-functional theory (DFT) optimization was performed in Gaussian09^21^ (B3LYP potential,^22,23^ def2TZVPP basis set,^24^ Grimme’s three-dimensional (3D) dispersion with the Becke–Johnson damping^25,26^). Visualization was performed in VESTA.^27^

## 3 Results

### 3.1 Phase Sequence in the 170–400 K Range

**Figure 2:**
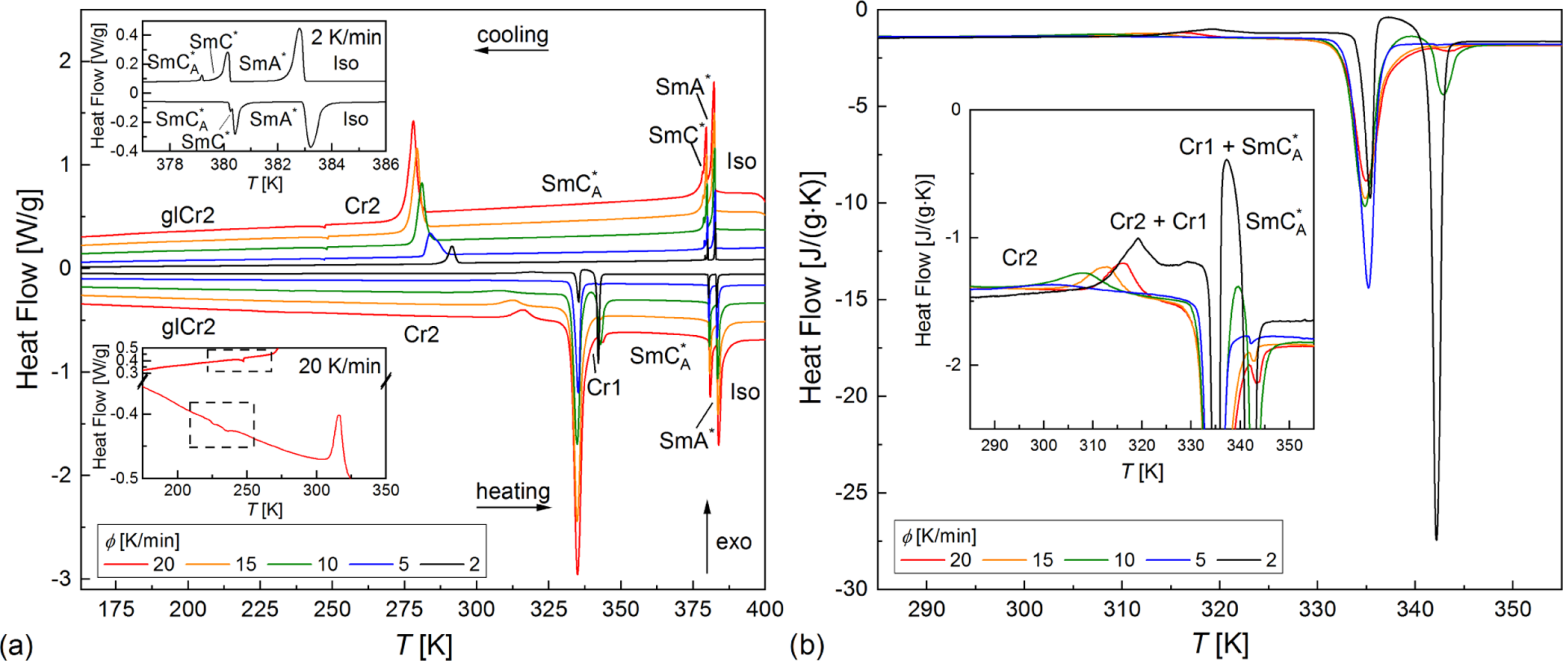
(a) DSC curves of 3F5FPhH6 collected during cooling and subsequent heating. The upper inset shows the transitions between the smectic phases. The bottom inset shows the Cr2/glCr2 transition. (b) DSC curves during heating, scaled by the heating rate. The inset shows the region of the recrystallization of Cr2, Cr2 → Cr1 transition and melting of the crystal phases.

**Figure 3:**
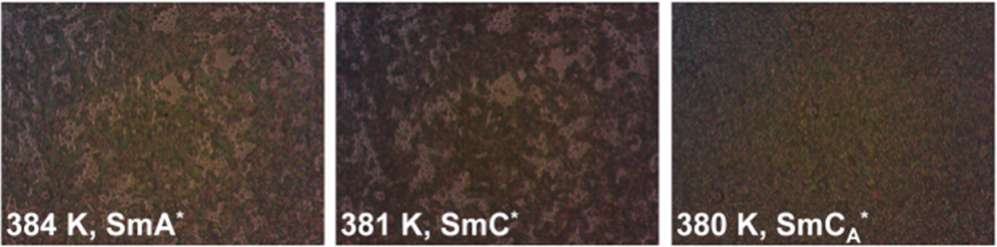
POM textures of the smectic phases of 3F5FPhH6 collected during cooling at 20 K/min.

**Figure 4:**
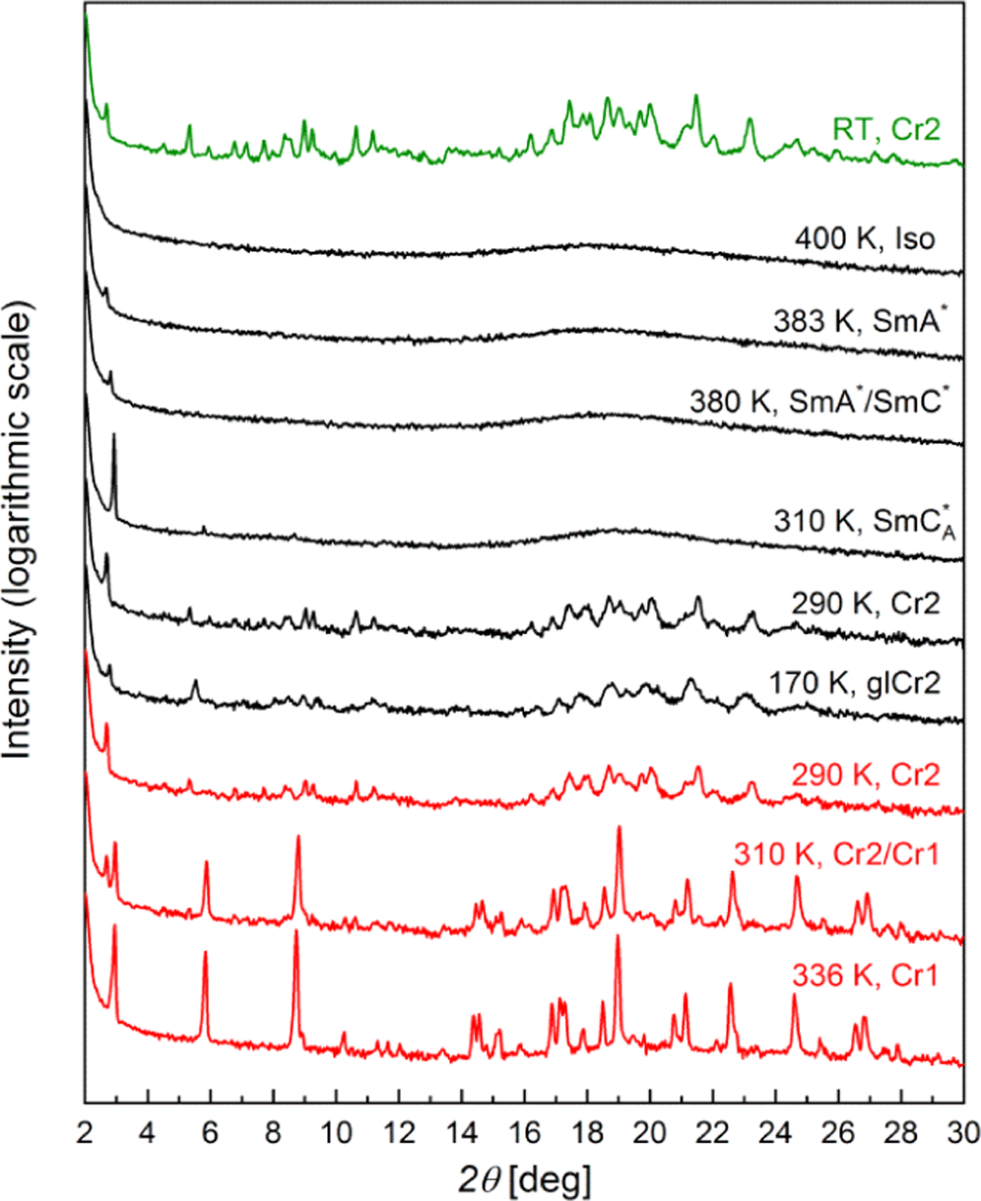
XRD patterns of 3F5FPhH6 collected at room temperature, on cooling from the isotropic liquid and on subsequent heating (the order of measurements is from top to bottom).

**Figure 5:**
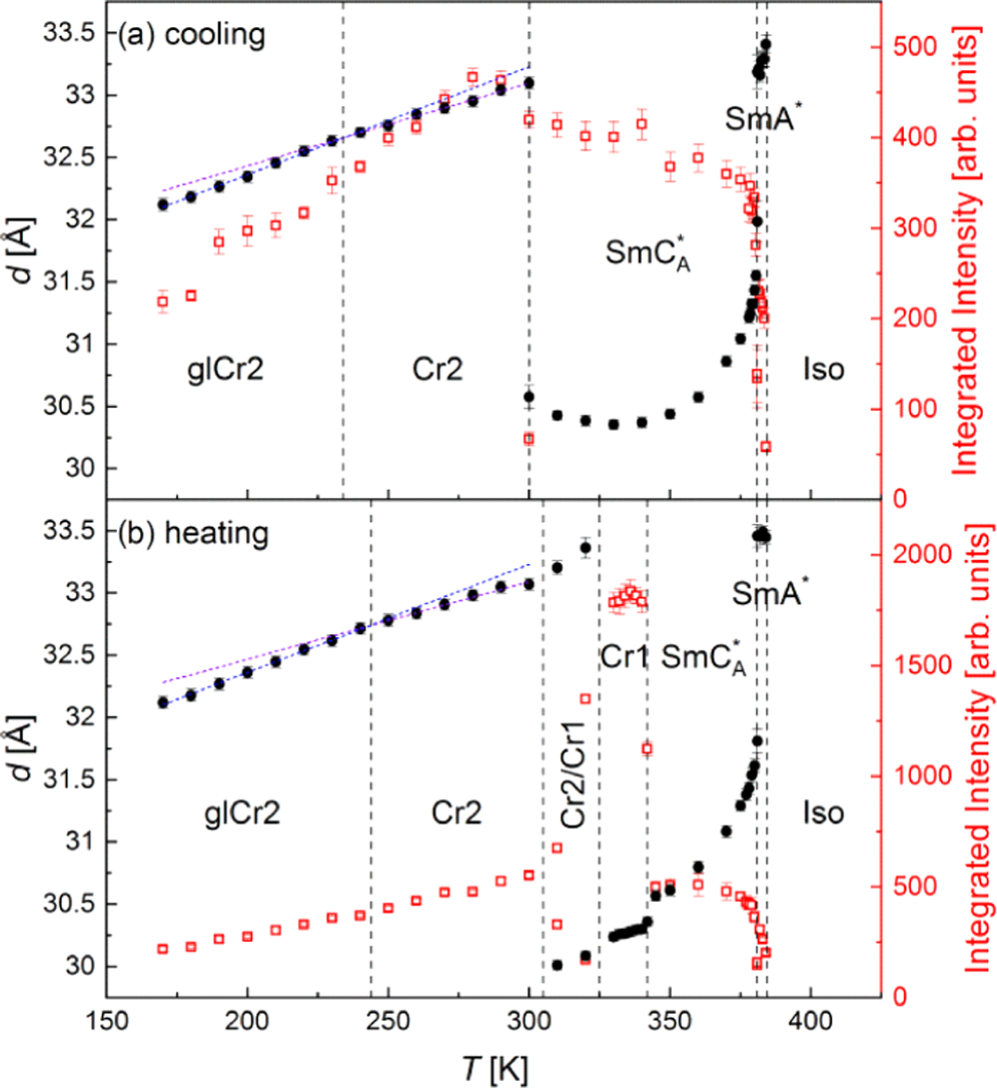
Smectic layer spacing/interplanar distance in crystal phases (left axis) and integrated intensity of the low-angle peak determined from the XRD patterns of 3F5FPhH6 on cooling (a) and heating (b). The SmC*/S*m*C_A_* transition was not visible in the XRD results; therefore, it is not indicated.

The DSC (Figure 2) and POM results (Figure 3) show that 3F5FPhH6 exhibits three smectic phases with the sequence SmA* → SmC* → SmC_A_* with decreasing temperature, in accord with the previous results.^1,2^ The X-ray diffraction patterns of these smectic phases (Figure 4) are characterized by the sharp peak in the low-angle region, at 2θ = 2.7–3.0°, which arises from the smectic layer order. At 2θ ≈ 18–20°, there is a wide, diffuse maximum arising from the short-range order within the smectic layers.^28^ The XRD pattern of the isotropic liquid also contains the diffuse maximum, but the low-angle peak is absent. As the temperature decreases, the low-angle peak from the XRD patterns of the smectic phases shifts toward higher 2θ values, indicating the decrease of the smectic layer spacing *d*, related to the peak’s position θ_d_ by the Bragg equation *d* = λ/2 sin θ_d_ (λ is the wavelength of the characteristic Cu Kα radiation).^28^ At the SmA*/SmC* transition at 381 K, one can see the coexistence of these phases, implying the first-order transition (Figure 5). The average layer spacing in the SmA* phase is 33.3(1) Å, which is 88% of the molecular length of 37.7 Å (defined as the distance between the terminal F and C atoms) obtained via DFT calculations for the extended 3F5FPhH6 molecule presented in Figure 1. When one considers the 3F5FPhH6 molecule with the nonchiral terminal chain in a gauche conformation (φ_4_ = 59°, φ_4_^’^ = −72° or φ_4_ = −59°, φ_4_^’^ = 72°), the molecular length is smaller, 36.6–36.7 Å, and the layer spacing in the SmA* phase is equal to 91% of the molecular length. The layer shrinkage at the SmA*/SmC* transition, defined as (*d*SmA* – *d*SmC*)/*d*SmA*, is 3.6(9)%. The XRD results show therefore that the SmA* phase of 3F5FPhH6 is a conventional orthogonal phase, not the de Vries phase where the molecules are actually tilted and the layer shrinkage is below 1%.^29^ The SmC* phase is present only in a very narrow temperature range (see the upper inset in Figure 2a), and the SmC*/S*m*C_A_* transition is not visible in the XRD results. In the S*m*C_A_* phase, the layer spacing decreases with the decreasing temperature down to a minimum of 30.4 Å at ca. 330 K (Figure 5b). On further cooling, the layer spacing increases to 30.6 Å at 300 K, where the crystallization begins.

The crystallization of 3F5FPhH6 occurs for all cooling rates up to 20 K/min, which is indicated by an exothermic anomaly in the DSC curve with an onset of 280–293 K, decreasing with the increasing cooling rate (Figure 2). In the XRD measurements, crystallization is observed at even higher temperature, 300 K, because the collection of the diffraction pattern in the 2–30° range lasted 24 min and during that time the sample was kept in isothermal conditions. The crystal phase formed during melt crystallization is denoted as Cr2. As the XRD patterns prove, it is the same crystal phase as observed after keeping the sample for a week at room temperature (Figure 4). The characteristic low-angle peak of the Cr2 phase is located at 2θ = 2.7–2.8°, which corresponds to the distance of 32–33 Å (Figure 5). It is between the layer spacing in the orthogonal SmA* and tilted SmC*, SmC_A_* phases; therefore, the Cr2 phase has probably a lamellar structure. On further cooling to 170 K, no transition between the crystal phases is observed; however, there is a kink in the DSC curve at 247 K that indicates the vitrification of the crystal phase (Figure 2). As the 3F5FPhH6 molecule has two flexible terminal chains, the vitrification of Cr2 is related probably to the freezing of the conformational disorder. During subsequent heating, the glass softening is observed at 236 K. In the XRD results, the glass transition of Cr2 is indicated by the change of the thermal expansion coefficient, as the slope of the *d*(*T*) dependence is smaller in the 240–300 K range than in the 170–240 K range (Figure 5). The glass-transition temperature *T*_g_, estimated at the intersection of fitted lines, is 234 K on cooling and 244 K on heating. The *T*_g_ values determined by DSC and XRD do not overlap precisely, but both methods indicate the region of the glass transition of Cr2 around 240 K.

**Figure 6:**
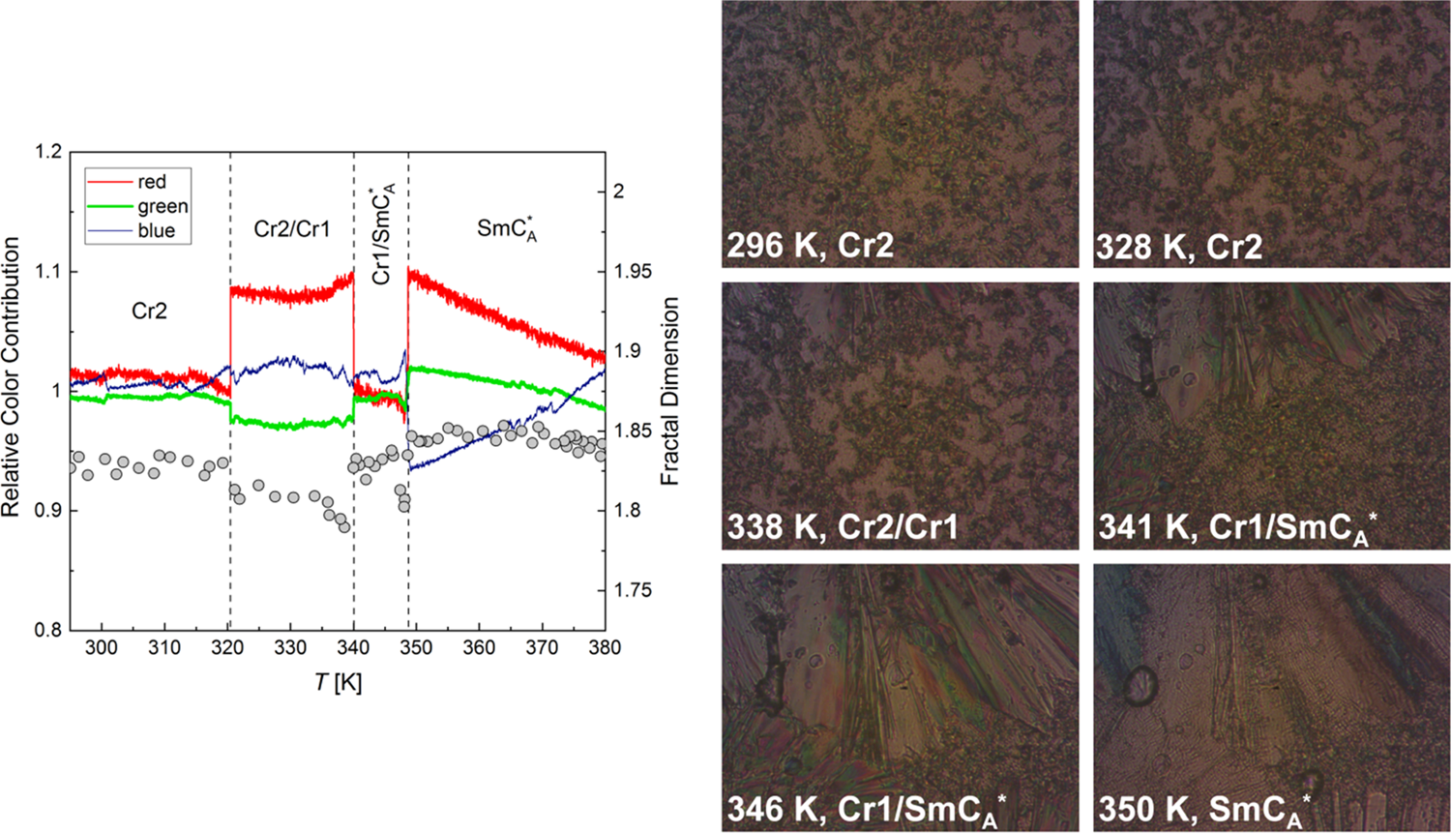
POM textures of 3F5FPhH6 collected during heating at 20 K/min and the results of the numerical analysis of textures in TOApy (relative color contribution, lines) and ImageJ (fractal dimension, points).

Another crystal phase of 3F5FPhH6, denoted as Cr1, is observed only on heating. In the XRD patterns, the development of the Cr1 phase is indicated by the appearance of the strong peak at 2θ = 3.0° at 310 K (Figure 4). The corresponding distance of 30.0–30.3 Å differs only by ca. 0.5 Å from the smectic layer spacing at the same temperature (Figure 5); therefore, Cr1 is presumed to have a lamellar structure, similarly to Cr2. The DSC results from heating in the temperature region of 290–350 K, where the Cr2 → Cr1 transition and melting of both crystal phases are observed, are shown in Figure 2b. The DSC curves in this figure were scaled by the heating rate to present more clearly the differences in the sizes of anomalies. For 2 K/min, the small exothermic anomaly with the onset at 313 K is interpreted as the Cr2 → Cr1 transition, as it corresponds to the XRD results. The exothermic anomaly is observed also during heating at 5–20 K/min (inset in Figure 2b); however, the onset temperature of this anomaly increases from 289 to 310 K and the area of the anomaly increases with increasing heating rate, which does not match the behavior of the exothermic anomaly observed for a 2 K/min heating rate. Thus, the exothermic anomaly at 5–20 K/min heating rates does not correspond to the Cr2 → Cr1 transition but instead to the recrystallization of Cr2.^30^ The degree of crystallinity of the Cr2 phase formed during cooling is expected to decrease with the increasing cooling rate; therefore, on subsequent heating with the same rate, the recrystallization is likely to have a larger energy effect, which is in accord with the increasing area of the exothermic anomaly for 5–20 K/min. At further heating, two endothermic anomalies are visible with the onset temperatures of 334 and 341.5 K for 2 K/min, corresponding to the Cr2 → SmC_A_* and Cr1 → SmC_A_* transitions, respectively. The relative sizes of these anomalies do not change monotonously with the increasing heating rate: the amount of Cr1 is the largest for 2 and 10 K/min (judging from the largest size of the anomaly at 341.5 K), while for the intermediate 5 K/min rate, the Cr1 → SmC_A_* transition is hardly visible in the DSC curve, similarly as for 15 and 20 K/min. The small fraction of the Cr1 phase for 5, 15, and 20 K/min confirms that the exothermic anomaly observed at these rates at lower temperature stems from the recrystallization of Cr2, not from the Cr2 → Cr1 transition. However, there is a question why the fraction of Cr1 shows an irregular dependence on the heating rate. The most reasonable cause is a very low nucleation rate of Cr1, which is confirmed by POM observations (Figure 6).

The interpretation of the POM textures can be facilitated by numerical analysis. In this study, two methods were applied. The TOApy program uses the “rgb” algorithm, where each pixel is decomposed into red, green, and blue components with the intensity denoted by numbers from the 0–255 range, and then the normalized sum over pixels is calculated to obtain the overall contribution of red, green, and blue components to the whole texture.^15^ This algorithm is useful when the phase transitions show in the POM observations as changes in the texture’s color. Another algorithm implemented in TOApy is “gray”, where the texture is converted into grayscale; the intensity of each pixel is denoted by 0–255, and the normalized sum over all pixels is calculated to obtain the overall brightness of the texture.^15^ Herein, we apply both “rgb” and “gray” algorithms. The values plotted by lines in Figure 6, denoted as the relative color contribution, are ratios of each “rgb” contribution and the overall “gray” value obtained for each texture. Such a solution enables the elimination of noise, which can be caused, e.g., by slight vibrations of the sample. Another method of numerical analysis, performed in the ImageJ program,^17^ is the fractal count box method,^16^ which enables the determination of the fractal dimension of a texture (plotted as points in Figure 6) after the conversion to a black-and-white image. The texture collected at 296 K during heating at 20 K/min, after the previous cooling with the same rate, belongs to the Cr2 phase. The numerical analysis indicates the phase transition at 320 K. The texture collected at 328 K is very similar to that collected at 296 K, indicating that it is still the Cr2 phase and the observed change in color is caused by recrystallization. However, above 320 K, the Cr1 phase can be already formed, and in the texture at 338 K, one can see the front of Cr1 appearing at the top of the texture. The next transition indicated by the numerical analysis occurs at 340 K. The textures at 341 and 346 K show that the abrupt change in the texture is caused by the melting of Cr2, while the Cr1 phase covers the increasing fraction of the observed area until it melts at 348 K. The texture of SmC_A_* at 350 K differs from the texture of the same phase collected at 380 K during cooling. It stems from different alignments of the smectic domains after cooling from the isotropic liquid and after melting of a crystal (in the latter case, the shape of the melted Cr1 crystallites is preserved in the sample’s alignment). The phase transition temperatures determined by POM are shifted to higher values compared with the DSC results; also, the Cr2 → Cr1 transition is facilitated in a very thin POM sample due to the heterogeneous nucleation at the surface. Despite that, one can see that Cr2 develops in the form of numerous crystallites of small size, while for Cr1, a small number of crystallites of much larger size is observed, indicating the low nucleation rate of Cr1 as it was previously presumed.

### 3.2 Crystallization Kinetics

#### 3.2.1 Nonisothermal Conditions

**Figure 7:**
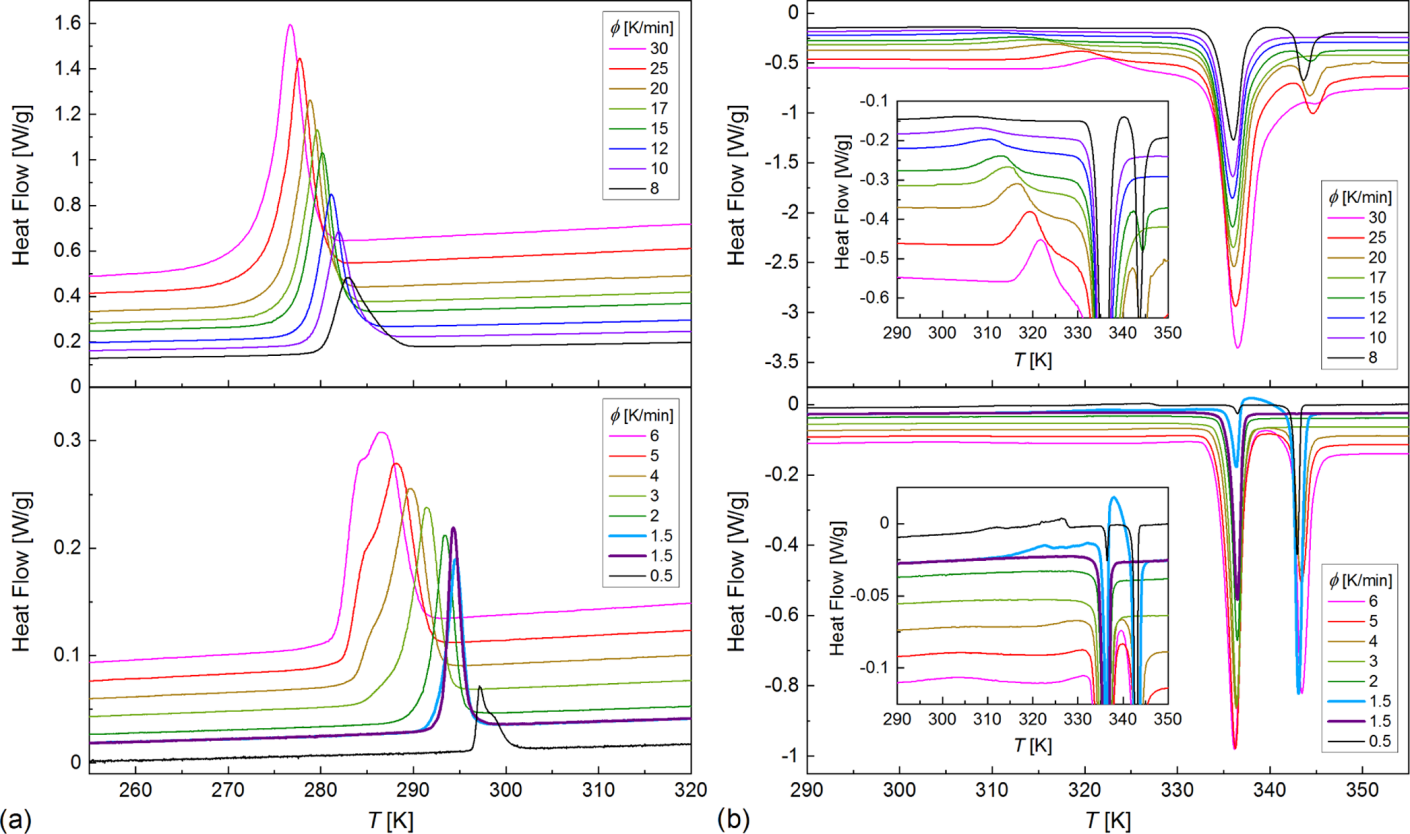
DSC curves collected during cooling (a) and subsequent heating (b) in the 228–363 K range. Insets in panel (b) are the enlarged parts of the DSC curves from the main panels. Results for 8–30 K/min (upper panels) and 0.5–6 K/min (bottom panels) are shown separately for clarity.

The DSC curves of 3F5FPhF6 from Figure 2a indicate the change in the crystallization kinetics for the 5 K/min cooling rate, where two overlapping endothermic anomalies are visible in the 275–290 K range. To investigate it in more detail, 15 different cooling/heating rates ϕ from 0.5 to 30 K/min were applied for additional DSC measurements in the 228–363 K range. The double anomaly corresponding to crystallization is clearly visible in the DSC curves registered on cooling at 0.5 and 4–6 K/min (Figure 7a), which means that the crystallization mechanism may change twice. To analyze such a complicated crystallization process, Friedman’s isoconversional method was applied,^31,32^ which involves the crystallization degree *X*, the temperature *T*_X_ where the given crystallization degree is obtained, and the effective activation energy *E*_eff_ at this temperature
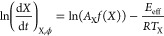
1*A*_X_ is the fitting parameter and *f*(*X*) stands for the reaction model, but its knowledge is not necessary for the determination of *E*_eff_, which is obtained from the slope of the ln(d*X*/d*t*) vs 1000/*T_X_* plot. The crystallization degree vs temperature for each ϕ was calculated from the DSC curves (Figures 2a and 7a) as^33^
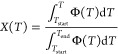
2where *T*_start_ and *T*_end_ stand for the temperature of the beginning and end of crystallization, respectively. Next, the crystallization rate was calculated by the differentiation of *X*(*T*) over time to be used in the ln(d*X*/d*t*) vs 1000/*T*_X_ plot for *X* = 0.1, 0.2, ..., 0.9 and for each cooling rate.

**Figure 8:**
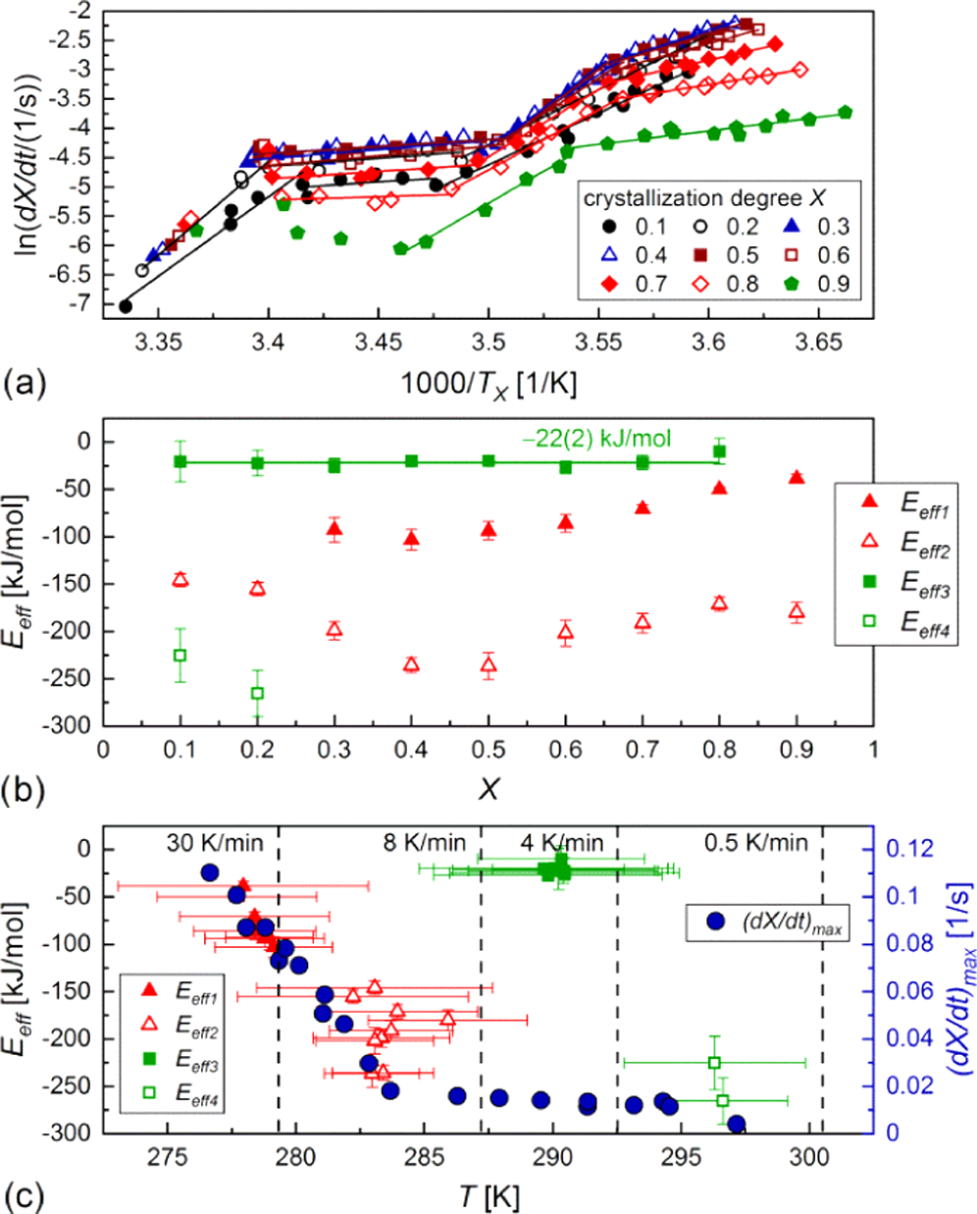
Nonisothermal melt crystallization of 3F5FPhH6 analyzed by Friedman’s isoconversional method: ln(d*X*/d*t*) vs inverted temperature *T*_X_ for various *X* (a) as well as *E*_eff_ vs *X* (b) and vs the average temperature where a given *X* is reached (c). The maximum d*X*/d*t* rates vs peak temperature in the DSC anomalies are also plotted in panel (c). The horizontal bars in panel (c) denote the temperature ranges, and the vertical lines indicate the onset temperatures of crystallization.

The results of the analysis by the isoconversional method are presented in Figure 8. The plot of ln (d*X*/d*t*) vs 1000/*T*_*X*_ (Figure 8a) is divided into four linear regions with different slopes, indicating different effective activation energies denoted as *E*_eff1_, *E*_eff2_, *E*_eff3_, and *E*_eff4_ in the order of increasing temperature. The border temperatures between these regions change with the crystallization degree *X*. The *E*_eff_ values also show various dependences on *X* (Figure 8b): *E*_eff1_ increases with increasing *X*, *E*_eff2_ has a minimum for intermediate *X* = 0.4–0.5, *E*_eff3_ is almost constant, while *E*_*eff*4_ was determined only for the beginning stages of crystallization during slow cooling. The effective activation energy is always negative. It means that the crystallization rate depends mainly on the nucleation rate and it increases with decreasing temperature, which is typical for melt crystallization, that occurs on cooling (for cold crystallization, which occurs on heating of the previously supercooled substance, the activation energy is usually positive and the crystallization rate increases with increasing temperature together with the diffusion rate).^32,34−37^ It is possible to plot *E*_eff_ vs temperature if one takes the average temperature corresponding to the experimental points used to determine a given *E*_eff_ value^32,37^ (Figure 8c). It is important to note that the horizontal bars in Figure 8c are not the error bars and instead they indicate the temperature range to which the particular *E*_eff_ value applies. The *E*_*eff*_(*T*) plot enables the easier interpretation of the crystallization process of 3F5FPhH6, especially when it is plotted together with the maximal crystallization rate corresponding to the peak temperature of the exothermic anomaly in the DSC curve. In the temperature region overlapping with crystallization at the lowest cooling rates (0.5–3 K/min), the effective activation energy is *E*_eff4_ = −(230–270) kJ/mol and the crystallization rate is expected to increase quickly with decreasing temperature. Indeed, the maximal crystallization rate (d*X*/d*t*)_max_ = 0.004 1/s for 0.5 K/min, while for 1.5 K/min, it is about three times larger, 0.011–0.014 1/s. For intermediate cooling rates of 4–6 K/min, (d*X*/d*t*)_max_ = 0.014–0.018 1/s, increasing weakly with decreasing temperature. It corresponds to the temperature range of *E*_eff3_, which shows the negative values closest to zero, −(10–30) kJ/mol. Crystallization during cooling at 8–30 K/min occurs in the temperature regions of *E*_eff2_ and *E*_eff1_, which seems to follow a common temperature dependence in the *E*_eff_(*T*) plot; thus, they govern the same crystallization mechanism. As *E*_eff2_ and *E*_eff1_ = −(40–240) kJ/mol, the (d*X*/d*t*)_max_ value increases significantly with increasing cooling rate, from 0.03 1/s for 8 K/min to 0.11 1/s for 30 K/min. As the temperature decreases, *E*_eff2_ and *E*_eff1_ gradually approach zero (except from *E*_eff2_ for intermediate *X*), indicating that for 30 K/min, (d*X*/d*t*)_max_ is close to the peak value, which corresponds to *E*_eff_ = 0.^32,37^ In total, three mechanisms of nonisothermal melt crystallization are observed for 3F5FPhH6, governed by *E*_eff1_, *E*_eff2_ (I), *E*_eff3_ (II), and *E*_eff4_ (III). The temperature regions of these effective energies overlap; therefore, for some cooling rates, the crystallization may occur according to two mechanisms, depending on the crystallization degree.

The DSC curves collected during heating with the same rate as applied upon the previous cooling show the irregularity of the Cr2 → Cr1 transition (Figure 7b). For 8–30 K/min, one can see a small exothermic anomaly, which arises from the recrystallization of Cr2. The endothermic anomalies indicate that most of the sample is in the Cr2 phase, as the anomaly at 334 K (melting of Cr2) is much larger than the anomaly at 343 K (melting of Cr1); for some rates, the latter anomaly is not observed, which means that the Cr1 phase was not formed in a detectable amount. For 0.5–6 K/min, the recrystallization of Cr2 is hardly visible, while the amount of the Cr1 phase is usually larger. We would like to draw the reader’s attention to the results for 1.5 K/min. During the first heating at a 1.5 K/min rate, the Cr2 → Cr1 transition is visible as the exothermic anomaly with the onset at 317 K, which is confirmed by a large endothermic anomaly at 342.5 K from the melting of Cr1. Meanwhile, for the second heating at 1.5 K/min, neither the exothermic anomaly from the Cr2 → Cr1 transition nor the endothermic anomaly from the melting of Cr1 is visible. Comparing both results for 1.5 K/min, it can be concluded that the development of the Cr1 phase occurs via the cold crystallization even after the melting of Cr2, as the exothermic anomaly is visible between the endothermic anomalies at 336.5 and 342.5 K. Only for the lowest applied rate of 0.5 K/min the Cr2 → Cr1 transition, with the onset at 306 K, is almost completed, as the area of the anomaly at 336.5 K indicates that the fraction of Cr2 just below the melting temperature is less than 10%.

#### 3.2.2 Isothermal Conditions

**Figure 9:**
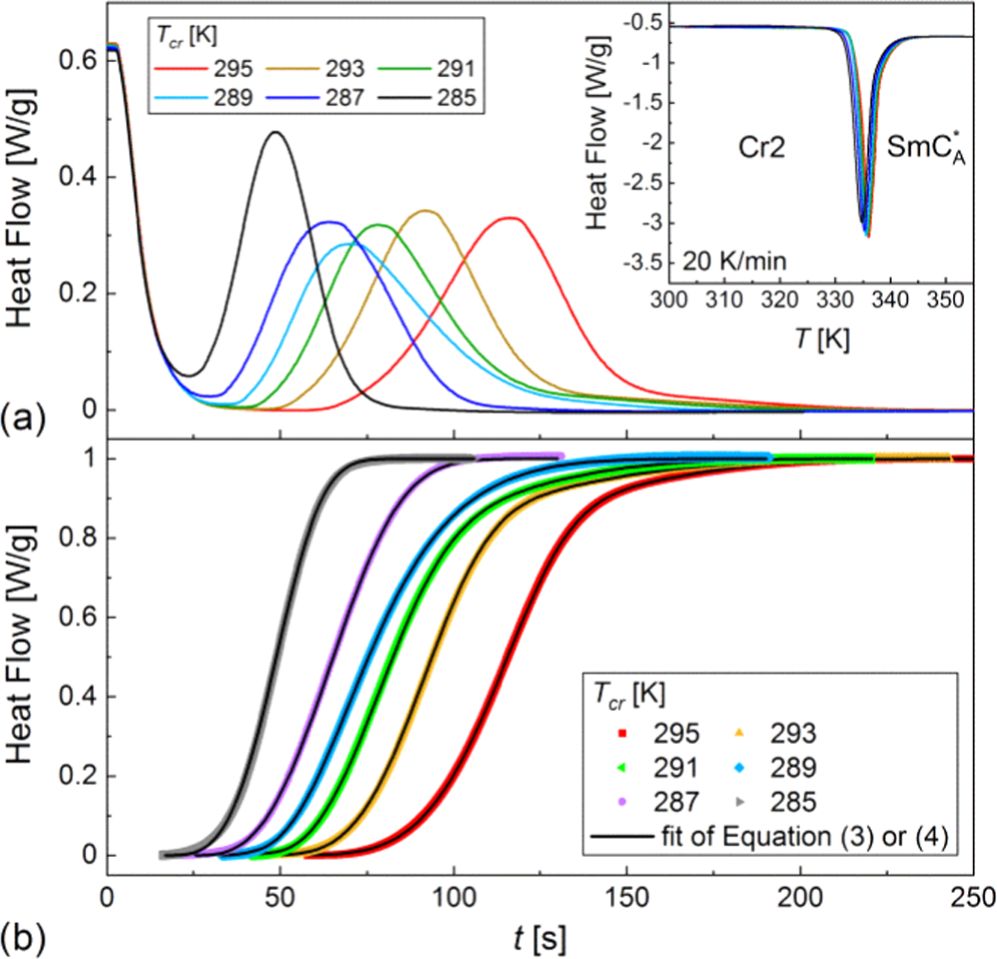
DSC curves collected during the isothermal crystallization of 3F5FPhH6 (a) with the corresponding crystallization degree vs time (b). The inset in panel (a) shows the DSC curves registered during heating after crystallization in each *T*_cr_.

**Figure 10:**
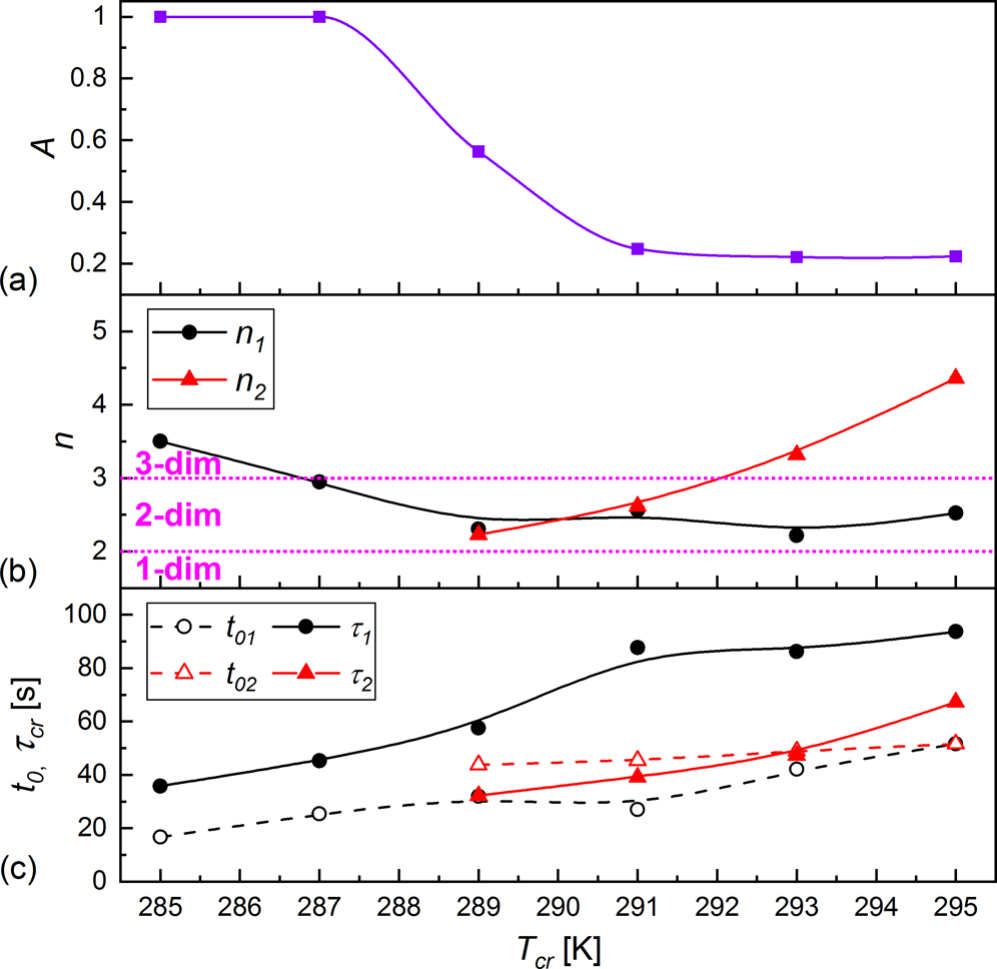
Parameters of the Avrami model determined by fitting eqs 3 or 4 to the experimental *X*(*t*) dependences for the isothermal crystallization of 3F5FPhH6: contribution of mechanism (1) (a), Avrami exponents (b), and initialization and characteristic times (c) vs *T*_cr_. The solid and dashed lines are a guide to the eye. The horizontal dotted lines in panel (b) denote the border *n* values for the 2-dimensional crystal growth. The uncertainties are smaller than symbol sizes.

The degree of isothermal melt crystallization vs time was calculated from the DSC curves collected after cooling the sample at 20 K/min to the selected crystallization temperature *T*_cr_ (Figure 9) using a formula resembling eq 2, where the integration was done over time between the initialization time *t*_0_ and time *t*_end_ when crystallization was completed. For all studied *T*_cr_ in the 285–295 K range, the developed crystal phase is Cr2 because it melts at 332–334 K (inset in Figure 9a). The model that enables the determination of the time scale of isothermal crystallization and supply information of the nucleation process and dimensionality of the crystal growth is the Avrami model^38−40^
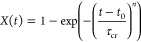
3The characteristic crystallization time τ_cr_ indicates the time after the beginning of crystallization when *X* = 1 – 1/*e* ≈ 0.63. The Avrami exponent *n* equals 1, 2, and 3 for the crystal growth in 1-, 2-, and 3-dimensions, respectively, with the assumption of the constant number of nuclei. When the nucleation rate is ≠0 and constant, then the corresponding Avrami exponent is larger, *n* = 2, 3, and 4, respectively.^40^ For the spherulitic growth, the *n* values can be even higher, equal to 5–6.^34^Equation 3 gives a good fit to the experimental *X*(*t*) dependences for crystallization in *T*_cr_ = 285 and 287 K, while for higher temperatures it is necessary to include two simultaneous crystallization processes described by different *t*_0_, τ_cr_, and *n* parameters. The two parallel crystallization processes are denoted as (1) and (2). Their initialization times follow the relation *t*_01_ < *t*_02_, except for *T*_cr_ = 295 K when it was necessary to introduce the constraint *t*_01_ = *t*_02_. If the sample’s fraction crystallizing according to mechanism (1) is denoted as *A*, the evolution of the total crystallization degree is described as^41^
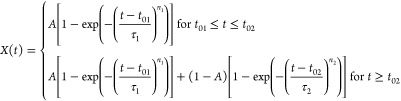
4The fitting results of eq 3 for *T*_cr_ = 285 and 287 K and eq 4 for *T*_cr_ = 289–295 K are shown in Figure 9b (for separate contributions of processes (1) and (2), see Figure S1 in the Supporting Information), and the fitting parameters vs *T*_cr_ are presented in Figure 10. The contribution of mechanism (1) is *A* = 1 for 285 and 287 K and only 0.22–0.25 in the 291–295 K range. The intermediate *A* = 0.56 value is obtained for *T*_cr_ = 289 K (Figure 10a), which is in the temperature region of 285–290 K where mechanisms (II) and (III) of nonisothermal crystallization overlap (Figure 8c). Mechanism (1) of crystallization is characterized by the Avrami parameter *n*_1_, which increases with increasing *T*_cr_: at 285 K, *n*_1_ = 3.5, which indicates the 3-dimensional crystal growth, while for 287–295 K, *n*_1_ is within the 2–3 range, indicating the 2-dimensional growth (Figure 10b). The Avrami parameter for mechanism (2) shows the inverse dependence, as it increases from *n*_2_ = 2.2 (2-dimensional growth) to 4.4 (3-dimensional growth) with increasing *T*_cr_ in the 289–295 K range. The initialization time *t*_01_ and characteristic time τ_1_ of the process (1) generally increase with increasing *T*_cr_. The same dependence is obtained for process (2), although the initialization time increases very slowly (Figure 10c). An increase in the crystallization time with increasing *T*_cr_ means that the crystallization rate is dependent more on the nucleation rate than the rate of diffusion between the SmC_A_* phase and Cr2 crystallites.^34^ The time scale of both crystallization processes is similar, and the total time necessary for the isothermal crystallization of 3F5FPhH6 is on the order of a few minutes in the studied *T*_cr_ range.

### 3.3 Dielectric Spectra

Various liquid crystalline phases are usually characterized by different relaxation processes, which enable phase identification. For the chiral smectic phases, these are collective relaxation processes, arising from the fluctuations of the tilt angle, which is the primary order parameter described by an amplitude (absolute value of tilt) and phase (azimuthal direction of tilt in the smectic layer plane).^28,42^ Fluctuations of the amplitude and phase of the tilt angle lead to two types of relaxation processes, amplitudons and phasons, respectively. Soft mode, an amplitudon, appears in the SmA* and SmC* phases. Its relaxation time and dielectric strength increase while approaching the SmA*/SmC* transition from both sides.^43,44^ The Goldstone mode, a phason, appears in the SmC* phase, and its frequency and strength are weakly dependent on temperature, except from the vicinity of the phase transition.^43,44^ In the SmC_A_* phase, there are two phasons: *P*_L_ at lower frequency and *P*_H_ at higher frequency, which are related, respectively, to in-phase and antiphase fluctuations of the tilt azimuth in the neighbor smectic layers.^45,46^

**Figure 11:**
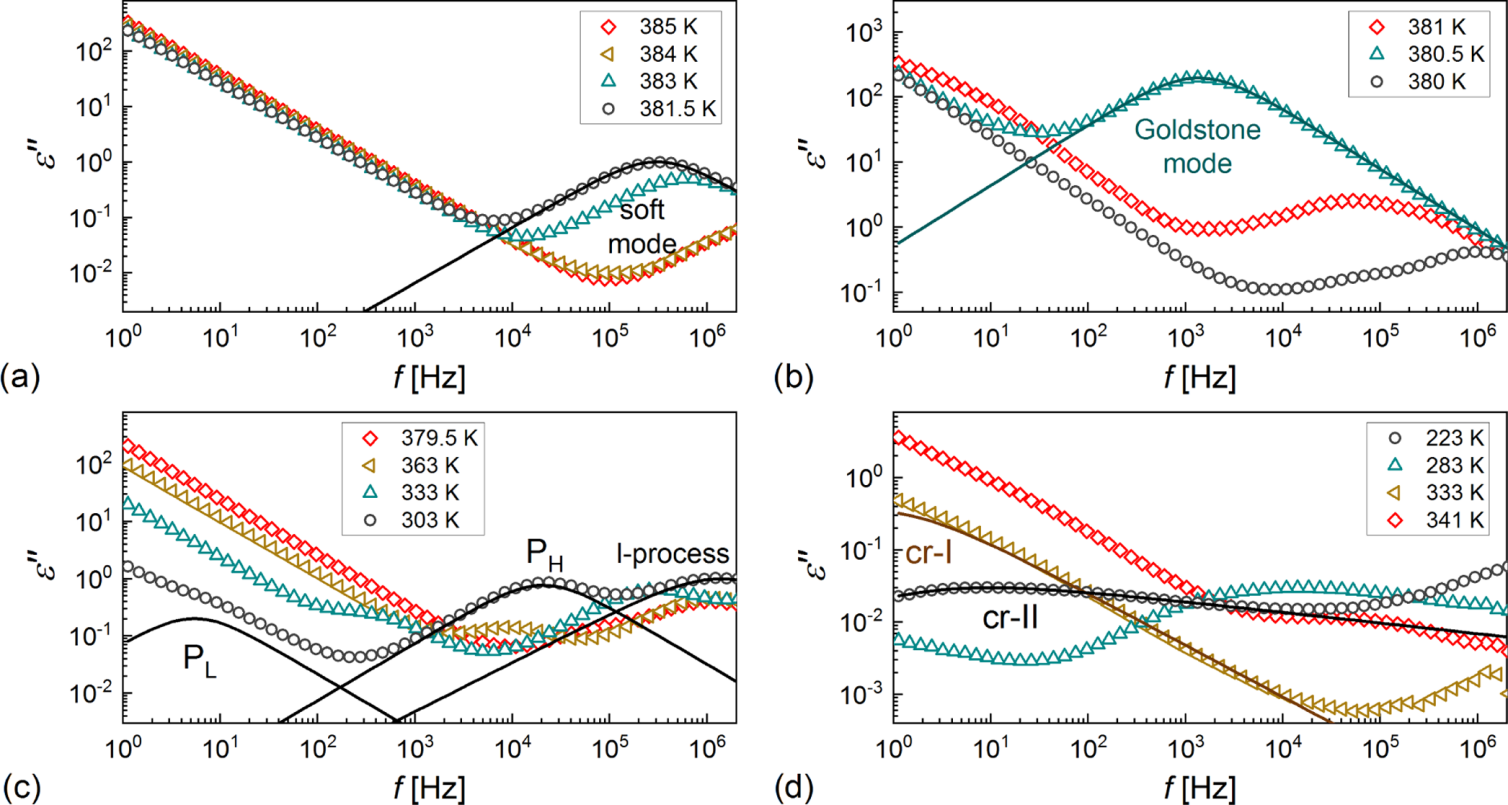
Dielectric absorption of 3F5FPhH6 vs frequency registered on cooling (a–c) and heating (d). The lines denote the fitting results of eq 5 separately for each relaxation process and with the omitted ionic conductivity contribution.

**Figure 12:**
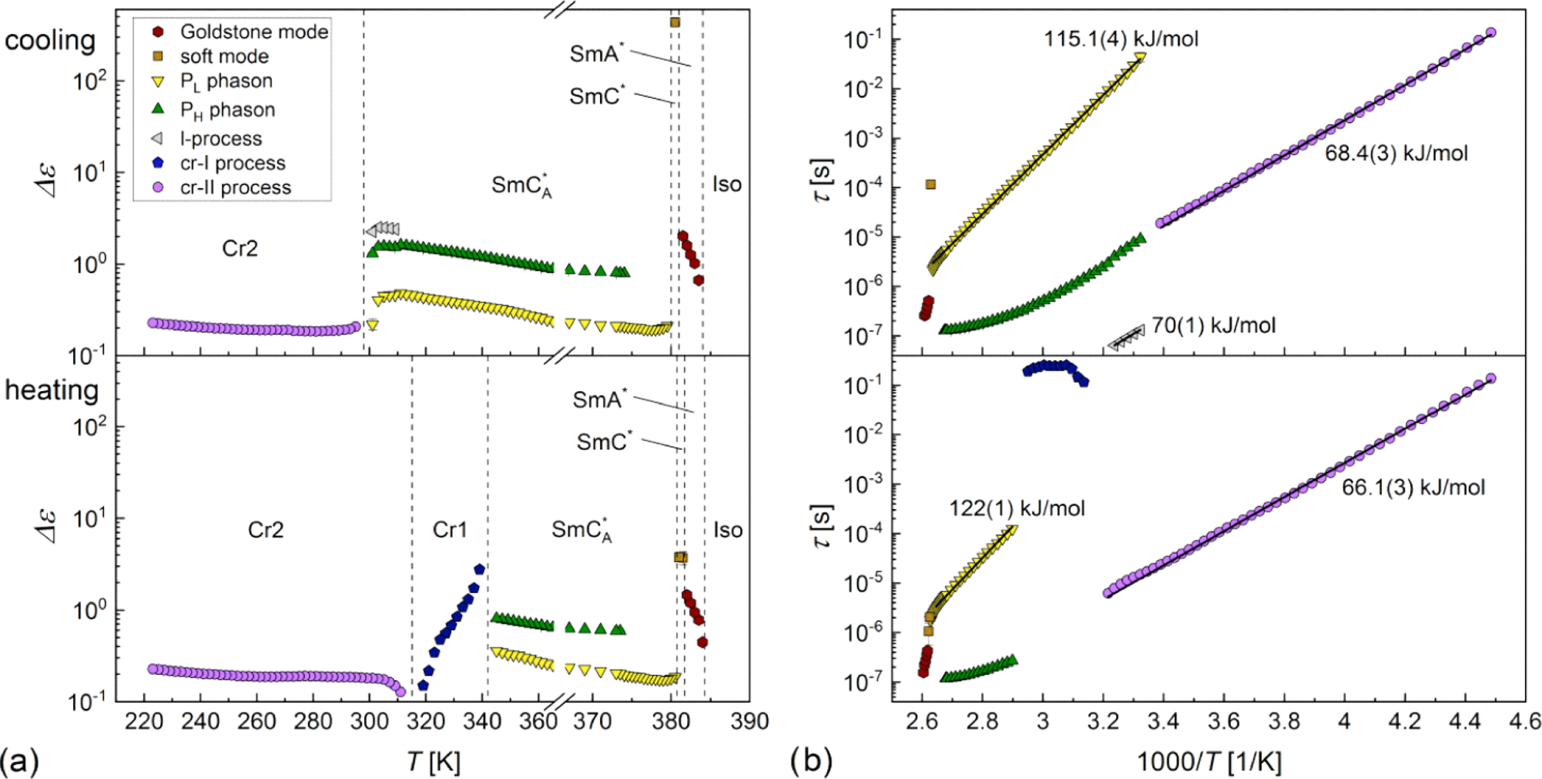
Dielectric strength vs temperature (a) and Arrhenius plot of relaxation times (b) of the relaxation processes of 3F5FPhH6 determined on cooling (upper panels) and heating (bottom panels). The legend in panel (a) applies to all panels.

The BDS spectra of 3F5FPhH6 are presented as two-dimensional (2D) maps in the Supporting Information in Figures S2 and S3. The dielectric strength Δε_*j*_ and relaxation time τ_*j*_ of the relaxation processes were determined by fitting the formula^42,47,48^
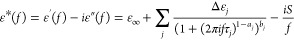
5where ε*(*f*) is the complex permittivity vs frequency, ε_∞_ is the permittivity for *f* → ∞, and *S* is the parameter describing the ionic conductivity. The *a*_*j*_ and *b*_*j*_ parameters describe the shape of the dielectric response. For *a*_*j*_ = 0 and *b*_*j*_ = 1, the relaxation process is of the Debye type; for *a*_*j*_ = (0,1) and *b*_*j*_ = 1, it is the Cole–Cole model^47^ and for *a*_*j*_ = (0,1) and *b*_*j*_ = (0,1) it is the Havriliak–Negami model.^48^ Representative experimental BDS spectra with the fitting results of eq 5, separately for each process, are shown in Figure 11. Although only the ε″(*f*) values are presented, the fitting of eq 5 was done simultaneously for the dispersion/real ε′(*f*) part and absorption/imaginary part ε″(*f*) of the permittivity. The Δε_*j*_ and τ_*j*_ values are presented in Figure 12.

#### 3.3.1 Relaxation Processes in the Smectic Phases

**Figure 13:**
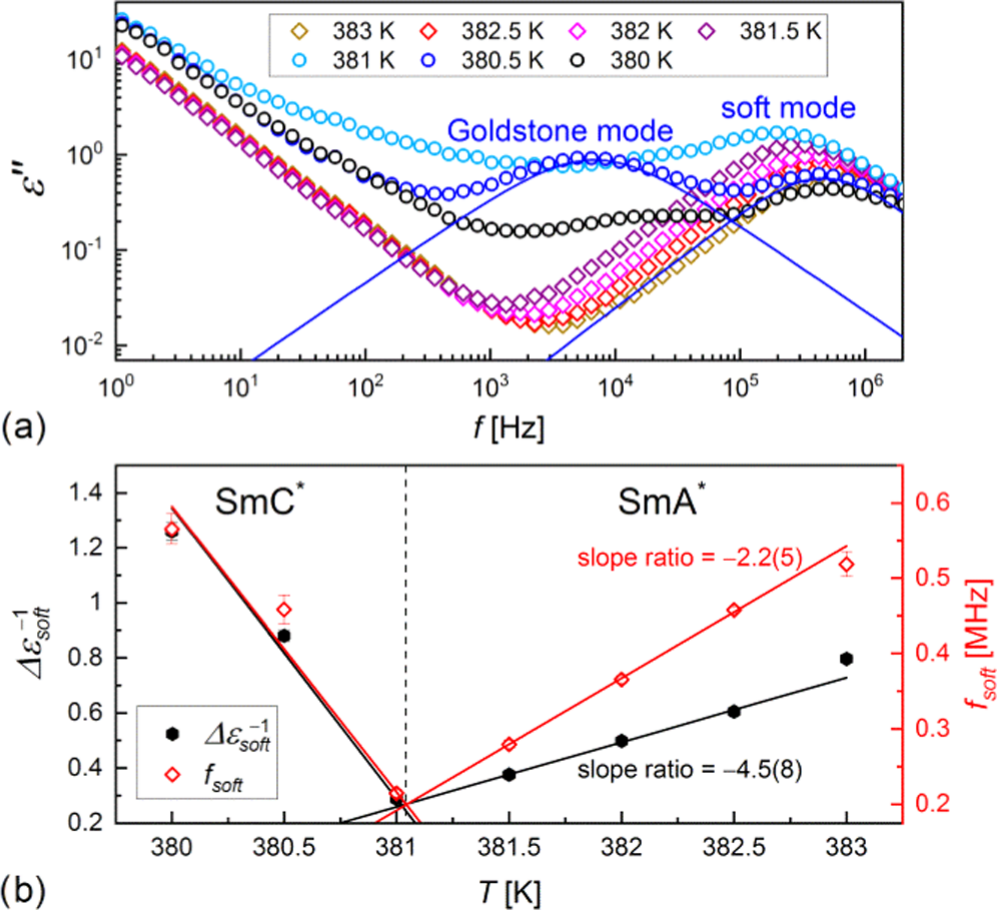
Dielectric absorption of 3F5FPhH6 in the SmA* and SmC* phases vs frequency registered on cooling in the 0.8 V/μm bias field (a) and the inverted dielectric strength and frequency of the soft mode vs temperature.

All processes in the smectic phases of 3F5FPhH6 are described by either Debye or Cole–Cole formulas. The identification of the liquid crystalline phases present in the narrow temperature range in the high-temperature region as SmA* and SmC* is confirmed by the observation of the soft mode and the Goldstone mode, respectively (Figure 11a,b). The Goldstone mode has the largest dielectric strength of all relaxation processes observed for 3F5FPhH6 (Figure 12a). Both the dielectric strength and relaxation time of the Goldstone mode are smaller on heating than on cooling, which is because of the change in the alignment of the sample during crystallization. The soft mode shows the characteristic increase of the dielectric strength and relaxation time with decreasing temperature in the SmA* phase. The soft mode also appears in the SmC* phase; however, it is covered by the stronger Goldstone mode. To suppress the Goldstone mode, one can use an external constant bias field.^42^ The BDS spectra collected in the 0.8 V/μm field are presented in Figure 13a. The soft mode is visible in both the SmA* and SmC* phases. The frequency *f*_soft_ = 1/2πτ_soft_ and the inverted dielectric increment Δε_soft_^–1^ decrease with a decreasing temperature in SmA* and increase in SmC* (Figure 13b), which is the dependence predicted for the soft mode.^43,44^ The ratio of slopes of the *f*_soft_(*T*) dependence in SmC* and SmA* is equal to −2.2(5), which agrees with the theoretical value of −2.^43,44^ For the Δε_soft_^–1^(*T*) dependence, the corresponding slope is −4.5(8). In the SmC_A_* phase, the in-phase phason *P*_L_ and antiphase phason *P*_H_ are observed (Figure 6c). The relaxation time of the *P*_L_ phason increases with a decreasing temperature in agreement with the Arrhenius formula τ(*T*) = τ_0_ exp(*E*_a_/*RT*), where *E*_a_ is an activation energy of 115.1(4) kJ/mol on cooling and 122(1) kJ/mol on heating (Figure 12b), which is connected with the overlapping of *P*_L_ with the molecular s-process (rotations around the short molecular axis).^45^ At 309 K, the third relaxation process at high frequencies enters the measured frequency range. This process is interpreted as the molecular l-process (rotations around the long molecular axis), and its activation energy is equal to 70(1) kJ/mol. The crystallization starts at 301 K; therefore, the l-process can be investigated only in a narrow temperature range. For glassforming 3F5HPhF6, 3F5HPhH6, and 3F5HPhH7 compounds, the τ(*T*) dependence of this process was determined for a much wider temperature range and the deviation from the Arrhenius formula was observed,^10,12,13^ indicating that it was the collective α-process.^34^ 3F5FPhH6 does not form the SmC_A_* glass; thus, the highest-frequency process in the SmC_A_* phase can be treated as a molecular process.

#### 3.3.2 Relaxation Processes in the Crystal Phases

In the Cr2 phase, a weak relaxation process denoted as cr-II is observed, which is described by the Havriliak–Negami formula due to the asymmetric shape of the absorption peak (Figure 11d). The relaxation time of the cr-II process shows an Arrhenius dependence on temperature, and the activation energy equals 68.4(3) kJ/mol on cooling and 66.1(3) kJ/mol on heating (Figure 12b). The width parameter *a* of the absorption peak increases in the 0.29–0.37 range, and the asymmetry parameter *b* decreases in the 0.23–0.30 range with a decreasing temperature. The overall shape of the absorption peak becomes wider and more asymmetric as the temperature decreases, which indicates the decreasing long-range interaction and increasing short-range interactions.^49^ In the Arrhenius plot, the cr-II process seems to be the continuation of the *P*_H_ process but it is rather only a coincidence because *P*_H_ is a process characteristic of the SmC_A_* phase. The activation energies of the cr-II process and l-process are similar but it does not indicate that they have the same origin. The XRD pattern of Cr2 (Figure 4) does not resemble typical diffraction patterns of crystal smectic phases, where rotations of the whole molecules would be possible.^50^ The cr-II process can be attributed instead to intramolecular rotations, making Cr2 the conformationally disordered (CONDIS) phase.^51,52^ The cr-II process diminishes on heating during the Cr2 → Cr1 transition (Figure 12a). In the Cr1 phase, the cr-I process arises with a very low frequency (Figure 11d). The dielectric strength and relaxation time of the cr-I process were estimated by fitting the Cole–Cole formula. The Δε values increase with increasing temperature, while the τ values, in the range of 0.12–0.26 s, do not show a monotonous dependence on temperature. Judging from the disappearance of the cr-II process, Cr1 is not a CONDIS crystal phase; therefore, the cr-I process is probably only the effect of the electrode polarization.^53^

#### 3.3.3 DFT Calculations

**Figure 14:**
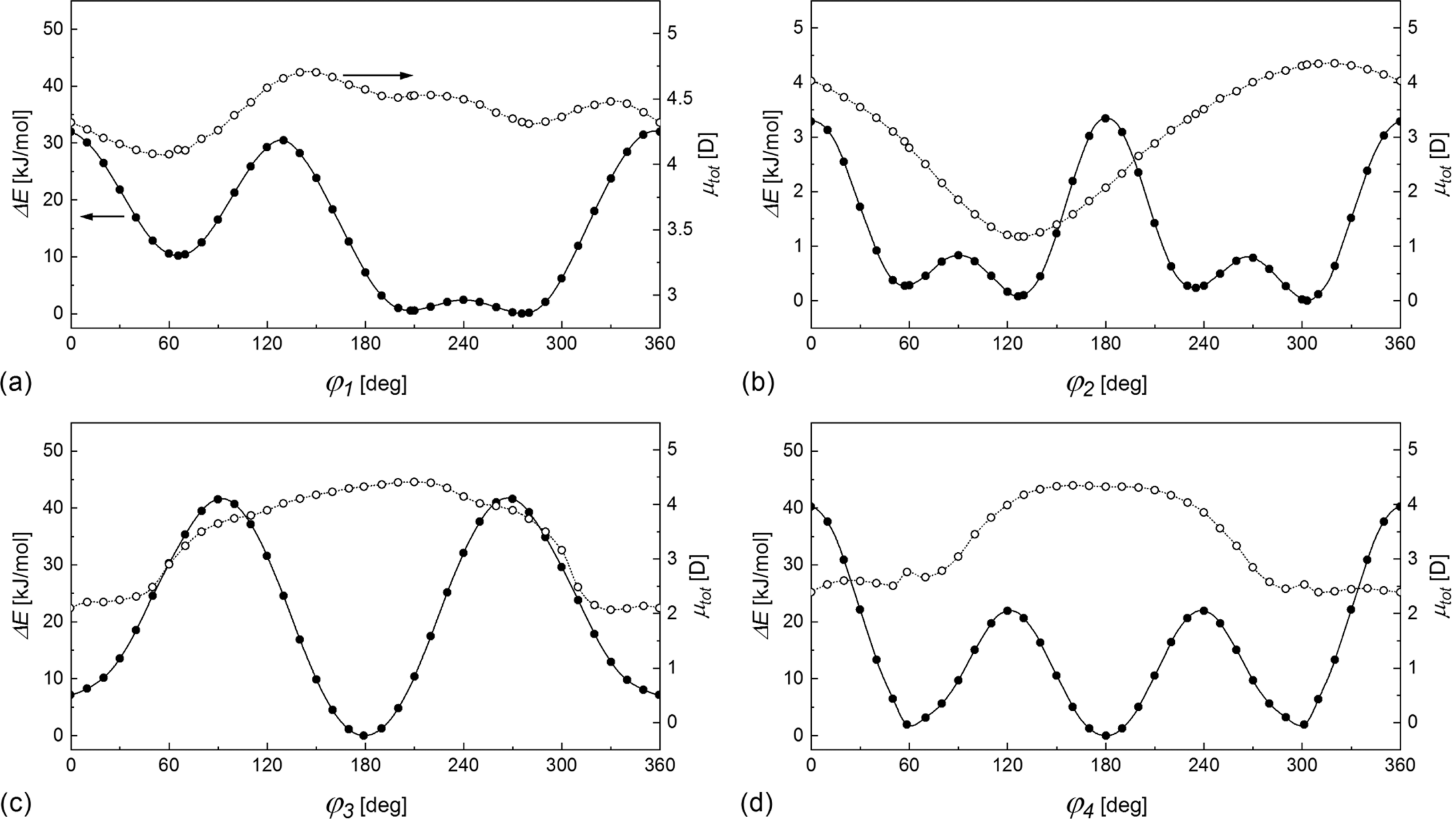
Conformational energy (solid symbols, left axis) and total dipole moment (open symbols, right axis) vs torsional angle for selected intramolecular rotations calculated for the isolated 3F5FPhH6 molecule with the DFT-B3LYP/TZVPP method. The φ_1_, φ_2_, φ_3_ (φ_3_ + φ_3_^’^ = 180°), and φ_4_ (φ_4_ = −φ_4_^’^) torsional angles are defined in Figure 1. The lines connecting the points are a guide to the eye. The preliminary results obtained by the semiempirical PM7 method are presented in Figure S4 in the Supporting Information.

The DFT-B3LYP/TZVPP calculations were performed for selected intramolecular rotations in the isolated 3F5FPhH6 molecule (Figure 14). The obtained energy barriers for particular torsional angles do not exceed 50 kJ/mol; therefore, it is necessary to consider at least two simultaneous rotations to explain the origin of the cr-II process, which has an activation energy of 66–68 kJ/mol. The rotation of the biphenyl can occur via the change of the (O=C)–O–C*–C torsional angle φ_1_ (Figure 14a; energy barrier 32 kJ/mol) simultaneously with the change of the (O=C)–O–C–C torsional angle φ_2_ between the −COO– group and biphenyl (Figure 14b; energy barrier 3.3 kJ/mol). The total conformational energy barrier is then ca. 35 kJ/mol. The rotation of biphenyl can occur also via the simultaneous change of φ_1_ and the C–C–C=O angle φ_3_ (energy barrier 33 kJ/mol), which would give the total energy barrier of 65 kJ/mol, close to the experimental activation energy of the cr-II process. The differences in the total dipole moment upon the change of φ_1_ are smaller than 0.7 D, while changes of φ_2_ and φ_3_ lead to the total dipole moment change of 3.2 and 1.1 D, respectively. Since the dielectric strength of the cr-II process is very low (Figure 12a), the latter situation is more probable. Rotation of the fluorinated benzene ring occurs via the change of φ_3_ and the C–O–C–C φ_3_^’^ angle with the φ_3_ + φ_3_^’^ = 180° constraint, and the energy barrier for this rotation is 41.5 kJ/mol (Figure 14c), too small to explain the cr-II process. Transitions between synperiplanar and gauche conformations in the terminal chains are unlikely to contribute to the relaxation processes observed in the crystal phases, as the exemplary energy barrier, calculated here for the −CF_2_–CH_2_–CH_2_–O– fragment (Figure 14d), is equal to only 22 kJ/mol. There is also another energy barrier of 40 kJ/mol but the conformational change between two minima of energy is more probable to occur via the smaller energy barrier. The DFT calculations imply that the origin of the cr-II process is the rotation of biphenyl via the simultaneous change of φ_1_ and φ_3_.

#### 3.3.4 Scaling of Dielectric Response

**Figure 15:**
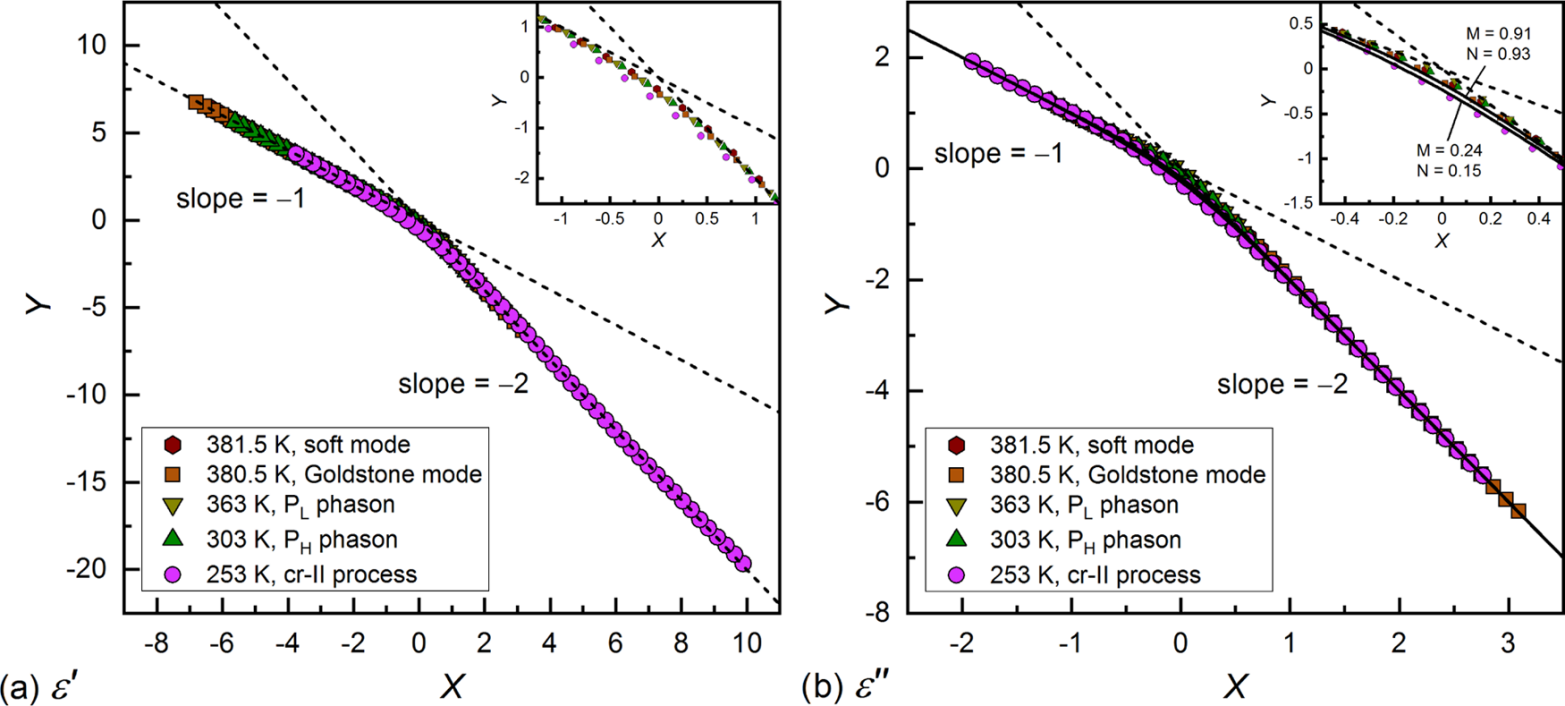
Scaling of the real part (a) and imaginary part (b) of the dielectric response of 3F5FPhH6 based on eq 6.^54,56,57^ The solid lines in panel (b) were calculated based on the Dissado–Hill cluster model^58^ for the border *M*, *N* values for ε″. The insets show the close-up to the kink in the master curve in each panel.

The real ε′(*f*) and imaginary ε″(*f*) parts of the complex dielectric permittivity show certain power laws, namely, ε′(*f*) – ε_∞_ ∝ *f*^0^ and ε″(*f*) ∝ *f*^M^ at the low-frequency side and (ε′(*f*) – ε_∞_) ∝ *f*^–*N*^ and ε″(*f*) ∝ *f*^–*N*^ at the high-frequency side.^54^ The *M*, *N* exponents, which should lay between 0 and 1 (*M*, *N* = 1 for a Debye process), can be determined from the slopes of the log–log plots of ε′(*f*) – ε_∞_ and ε″(*f*), as shown for 3F5FPhH6 in Figures S5 and S6 in the Supporting Information. The coordinates of the intersection points of these power laws are denoted as *f*_s_ and ε_s_.^55^ Using the *M*, *N*, *f*_s_, and ε_s_ parameters, it is possible to put the dielectric data for various relaxation processes on one master curve by making a plot of *Y*(*X*), where^54,56,57^
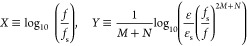
6The l-process in the SmC_A_* phase and the cr-I process in the Cr1 phase were not scaled because they were only partially visible in the BDS spectra. For the Goldstone mode in the SmC* phase and for *P*_L_ and *P*_H_ phasons in the SmC_A_* phase, the *N* parameter determined from ε′(*f*) – ε_∞_ exceeded 1, which is caused likely by overlapping of the relaxation processes, which makes it difficult to introduce the proper value of ε_∞_ (note that scaling is supposed to be done without fitting of any model like, e.g., Havriliak–Negami’s). Despite that, the scaling of ε′(*f*) – ε_∞_ using eq 6 was still applicable. The slope *N* > 1 for ε′(*f*) – ε_∞_ was obtained also for some other compounds.^54^ The *N* slope in the (0, 1) range was obtained for ε′(*f*) – ε_∞_ of single processes, like the soft mode in the SmA* phase and the cr-II process in the Cr2 phase. Meanwhile, for ε″(*f*), the *M*, *N* slopes in the (0,1) range were obtained even for overlapping processes. The master curves of ε′(*f*) – ε_∞_ and ε″(*f*) are presented in Figure 15a,b, respectively. The data from the low-frequency side and high-frequency side are located on the lines with the slopes of −1 and −2, respectively. The differences between particular processes are visible only at the kink in the master curve (insets in Figure 15a,b). The results for 3F5FPhH6 were compared with the Dissado–Hill cluster model,^58^ which is not an empirical formula like the Cole–Cole model or Havriliak–Negami model, but it is derived based on the interactions between dipoles. The Dissado–Hill model describes the dielectric permittivity as
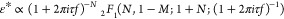
7where _2_*F*_1_ is the Gauss hypergeometric function. The ε″ values were calculated numerically^59^ with eq 7 using the border *M*, *N* values determined for 3F5FPhH6, namely, *M* = 0.91, *N* = 0.93 and *M* = 0.24, *N* = 0.15 and subsequently they were scaled according to eq 6. The scaled ε″ values calculated from the Dissado–Hill model are indicated by lines in Figure 15b. The experimental points near the kink are not situated exactly between the calculated lines, as was obtained for similar compounds.^13,60^ However, the deviations for 3F5FPhH6 are not significant, as they are of ca. 0.1 on the *Y* axis for the *P*_L_-process and of ca. 0.6 and smaller for other processes, and they are caused likely by the overlapping of processes in the BDS spectra with each other or with the conductivity contribution at low frequencies, which leads to bias in the *M*, *N* values.

## 4 Discussion

**Figure 16:**
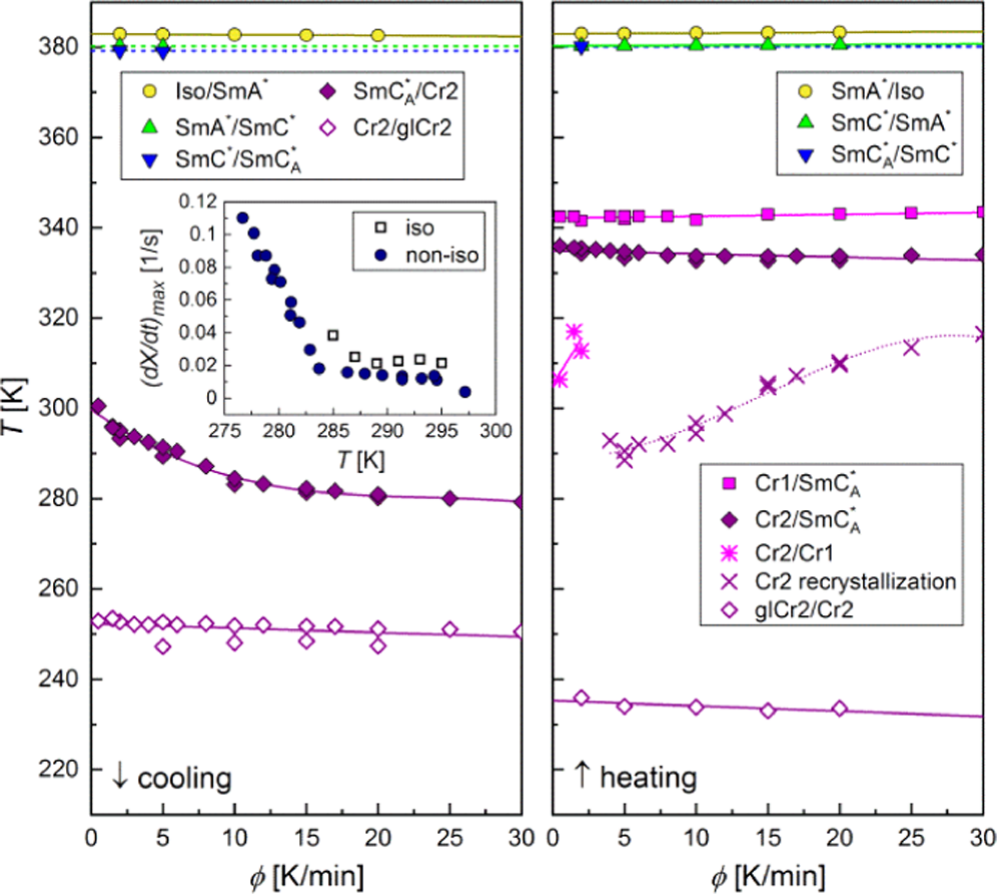
Phase transitions of 3F5FPhH6 for various cooling/heating rates, as determined from the DSC results. The inset shows the comparison of the maximal crystallization rate in isothermal and nonisothermal conditions.

The summary of the phase transitions of 3F5FPhH6 is presented in Figure 16 and Table 1. In the temperature range of 170–400 K, the 3F5FPhH6 compound shows the isotropic liquid phase, three chiral smectic phases (paraelectric SmA*, ferroelectric SmC*, antiferroelectric SmC_A_*), and two crystal phases (Cr2, Cr1). The polymorphism of the smectic phases is in agreement with the previous results,^1,2^ and the transition to the hexatic smectic phase, reported for 3F5HPhH6 and 3F5HPhH7,^12−14^ is not observed. Even for fast cooling with the 30 K/min rate, 3F5FPhH6 undergoes crystallization to the Cr2 phase and the SmC_A_* glass is not obtained. Cr2 is a conformationally disordered (CONDIS) phase, with the melting temperature *T*_*m*_ = 336 K and entropy of melting Δ*S*_m_ = 52 J/(mol·K). The relaxation process cr-II in this phase is identified as the rotation of the biphenyl in the molecular core based on its activation energy of 66–68 kJ/mol and DFT calculations. The DSC and XRD results imply the glass transition in the Cr2 phase to be around 240 K, which is interpreted as the freezing of conformational degrees of freedom. However, the relaxation time of the cr-II process is equal to ca. 0.01 s, around 240 K, well below 100 s, which is usually considered the point of the glass transition.^61^ It implies that the cr-II process is not involved in the glass transition of Cr2 and freezing of disorder is related to other intramolecular motions, which are not visible in the BDS spectra because they do not cause the detectable change in the dipole moment. The Cr1 phase of 3F5FPhH6 is characterized by *T*_m_ = 343 K and Δ*S*_m_ = 70 J/(mol·K). The entropy of melting is larger by 20 J/(mol·K) than for Cr2; therefore, Cr1 is unlikely to be the conformationally disordered phase. The cr-I process observed in the BDS spectra at low frequencies in the temperature range of Cr1 is caused probably by the electrode polarization. The Cr2 → Cr1 transition occurs only on heating. Due to a very low nucleation rate of Cr1, this transition is usually incomplete in the conditions of constant heating.

**Table 1:** Onset Temperatures *T*_o_, Peak Temperatures *T*_p_, Enthalpy Change Δ*H*, and Entropy Change Δ*S* = Δ*H*/*T*_p_ for the Phase Transitions of 3F5FPhH6 Determined from the DSC Curves Collected at 0.5–2 K/min Cooling/Heating Rates^a^

transition	*T*_o_ [K]	*T*_p_ [K]	Δ*H* [kJ/mol]	Δ*S* [kJ/mol]
Iso → SmA*	383.0	382.8	–4.1	–6.2
SmA* → SmC*	380.2	380.1	–1.4	–2.2
SmC* → SmC_A_*	379.2	379.2	–0.3	–0.5
SmC_A_* → Cr2	300.5	297.2	–11.1	–37.5
Cr2 → glCr2	253			
glCr2 → Cr2	236			
Cr2 → Cr1	306.4	326.4	–6.0	–18.4
Cr2 → SmC_A_*	335.6	336.5	17.6	52.2
Cr1 → SmC_A_*	342.5	343.0	24.7	72.0
SmC_A_* → SmC*	380.2	380.2	0.3	0.5
SmC* → SmA*	380.3	380.4	1.5	2.2
SmA* → Iso	383.0	383.2	4.0	6.1

a Positive and negative Δ*H* and Δ*S* values indicate an endothermic and exothermic transition, respectively.

The melt crystallization of 3F5FPhH6 is complex. The DSC results for both nonisothermal and isothermal crystallization imply some changes in the sample around 290 K. As was already mentioned, it cannot be explained by the transition to the monotropic hexatic smectic phase. The melt crystallization of 3F5FPhH6 is controlled mainly by the thermodynamic driving force, which can be determined as Δ*G*(*T*) ≈ (*T*_m_ – *T*)Δ*S*_m_.^62^ As the temperature decreases, Δ*G*(*T*) increases, which consequently increases the nucleation rate. Let us consider the thermal energy related to the translational degrees of freedom. In the Cr2 phase, the molecules have no translational degree of freedom. In the SmC_A_* phase, the molecules can move within a two-dimensional smectic layer, while the diffusion from layer to layer is strongly hindered;^28^ therefore, one can assume two translational degrees of freedom. According to the equipartition theorem, thermal energy related to two translational degrees of freedom equals 2·*RT*/2 = *RT*. The temperature where Δ*G*(*T*) for Cr2 and *RT* coincide is *T*_0_ = *T*_m_Δ*S*_m_/(*R* + Δ*S*_m_) = 289.5 K. Below this temperature, Δ*G*(*T*) exceeds the thermal energy of translational degrees of freedom in the SmC_A_* phase, which corresponds to the faster increase of the maximal crystallization rate (d*X*/d*t*)_max_ (inset in Figure 16). The hypothesis is that the diffusion of molecules within the smectic layers interrupts the formation of stable nuclei in the crystal phase; therefore, crystallization is facilitated when Δ*G*(*T*) > *RT*. It explains various mechanisms of nonisothermal crystallization (I) for *T* < 285 K, (II) for *T* = 285–295 K, and (III) for *T* > 295 K and of isothermal crystallization (1) for *T*_cr_ = 285–289 K and (2) for *T*_cr_ = 289–295 K. The maximal crystallization rate is lower for nonisothermal crystallization than for isothermal one at the same temperature. It is because during nonisothermal crystallization, the nucleation occurs at a higher temperature than the crystal growth, while the former usually has a peak rate at a lower temperature than the latter.^63^

The 3F5FPhH6 compound crystallizes easily on cooling, which differs it from the three 3F5X_1_PhX_2_6 counterparts with other types of fluorination of the benzene ring,^10−12^ with a high tendency to vitrification of the smectic phase. Considering the results for all 3F5X_1_PhX_2_6 compounds, it can be concluded that for *m* = 5 and *r* = 6, fluorosubstitution at the X_1_ position leads to easier crystallization, while fluorosubstitution at the X_2_ position increases the tendency to the formation of the smectic glass during cooling. The stability width of the SmC_A_* phase, defined as *T*_c_ – *T*_m_, where *T*_c_ is the temperature of the SmC*/SmC_A_* transition, corresponds well with the glassforming properties of the 3F5X_1_PhX_2_6 homologues, as it is the smallest for 3F5FPhH6 (43 and 36 K if one considers *T*_m_ of Cr2 and Cr1, respectively). For the glassforming 3F5X_1_PhX_2_6 compounds, the stability range is significantly wider, namely, 53 K for 3F5FPhF6,^11^ 55 K for 3F5HPhH6,^12^ and 68 K for 3F5HPhF6.^10^ Another contributing factor is the relaxation time of the *P*_H_ process in the SmC_A_* phase. In the recent publication,^64^ the hypothesis was given that slower *P*_H_ process enabled faster crystallization. The BDS results for the 3F*m*X_1_PhX_2_6 homologues are in agreement with this hypothesis. At 303 K (the lowest temperature before the beginning of crystallization of 3F5FPhH6), the relaxation time of the *P*_H_ process is larger for 3F5FPhH6 than for other 3F*m*X_1_PhX_2_6 counterparts.^10−12^ The glassforming 3F5HPhF4 compound^9^ with a shorter chiral chain (*r* = 4) also follows these rules, as it possesses a very wide stability range of SmC_A_* (88 K) and the relaxation time of the *P*_H_ process is shorter than for 3F5FPhH6. The exception is the glassforming 3F5HPhH7 compound^13,14^ with a longer chiral chain (*r* = 7), for which the relaxation time of the *P*_H_ process is longer; however, the stability range of the SmC_A_* phase (45 K) is still slightly wider than for 3F5FPhH6. Since the length of the chiral chain may have an additional influence on the crystallization kinetics, the comparison between the stability range and the molecular dynamics in the SmC_A_* phase should be considered mainly among compounds differing only by one detail of the molecular structure, in this case, the fluorosubstitution of the benzene ring.

## 5 Summary and Conclusions

The 3F5FPhH6 compound with a partially fluorinated terminal chain and benzene ring was investigated by differential scanning calorimetry, polarizing optical microscopy, X-ray diffraction, and broad-band dielectric spectroscopy. The presence of three chiral smectic phases, SmA*, SmC*, and SmC_A_*, was confirmed, and two crystal phases, low-temperature CONDIS Cr2 phase and high-temperature Cr1 phase, were reported. The relaxation processes observed in the BDS spectra of the smectic phases and Cr2 phase can be scaled to one master curve. 3F5FPhH6 is the only 3F5X_1_PhX_2_6 homologue that does not form the vitrified SmC_A_* phase, which is attributed to the most narrow stability range of SmC_A_* and the longest relaxation time of the *P*_H_ phason compared to the other three 3F5X_1_PhX_2_6 counterparts. The melt crystallization of 3F5FPhH6 is controlled mainly by nucleation, but the analysis with Friedman’s isoconversional method shows that the effective activation energy is dependent on temperature. The changes in the crystallization mechanism, in both isothermal and nonisothermal conditions, occur around 290 K. It corresponds to the temperature where the thermodynamic driving force (increasing with decreasing temperature) intersects with the thermal energy of two translational degrees of freedom in the SmC_A_* phase (decreasing with increasing temperature). The obtained results imply that both the *P*_H_ process and movements of molecules within the smectic layers slow down crystallization, which is caused probably by the hindering effect on the formation of stable nuclei of the crystal phase.

## Supporting Information Available

Fitting results of a double Avrami model (Figure S1); dielectric absorption vs frequency and temperature on cooling (Figure S2) and heating (Figure S3); results of molecular modeling with the PM7 method (Figure S4); and determination of the parameters of the scaling model of BDS spectra (Figures S5 and S6) (PDF)
