# Supplementary material for: Investigation of Chiral Smectic Phases and Conformationally Disordered Crystal Phases of the Liquid Crystalline 3F5FPhH6 Compound Partially Fluorinated at the Terminal Chain and Rigid Core
Source: J Phys Chem B. 2022 Aug 19;126(34):6547–61. doi: 10.1021/acs.jpcb.2c03654 (PMC9442646; doi:10.1021/acs.jpcb.2c03654)
Supplement: Supplementary file 1 — jp2c03654_si_001.pdf [file jp2c03654_si_001.pdf]

Investigation of Chiral Smectic Phases and Conformationally Disordered Crystal Phase of Liquid Crystalline 3F5FPhH6 Compound Partially Fluorinated at Terminal Chain and Rigid Core

Aleksandra Deptuch[1,*], Małgorzata Jasiurkowska-Delaporte[1], Ewa Juszyńska-Gałązka[1,2], Anna Drzewicz[1], Marcin Piwowarczyk[1], Magdalena Urbańska[3], Stanisław Baran[4]

[1] Institute of Nuclear Physics Polish Academy of Sciences, PL-31342 Kraków, Poland

[2] Research Center for Thermal and Entropic Science, Graduate School of Science, Osaka University, 560-0043 Osaka, Japan

[3] Institute of Chemistry, Military University of Technology, PL-00908 Warsaw, Poland

[4] M. Smoluchowski Institute of Physics, Jagiellonian University, PL-30348 Kraków, Poland

[*]corresponding author, aleksandra.deptuch@ifj.edu.pl

Supporting Information

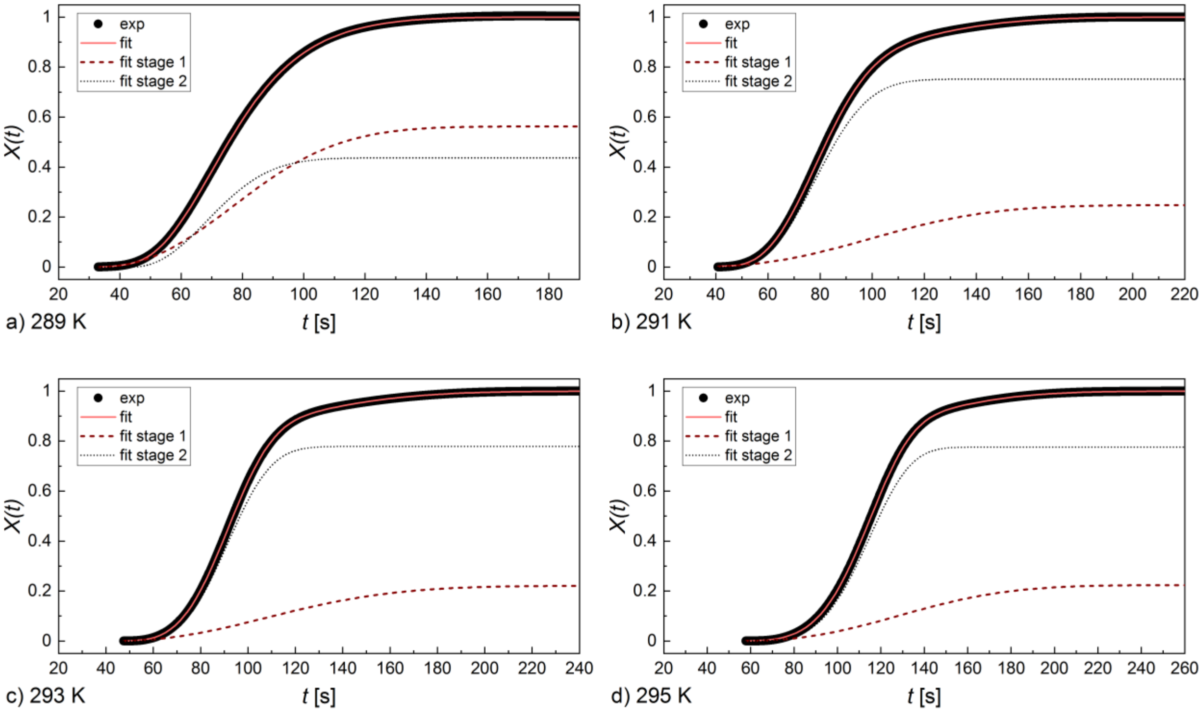

Figure S1. Fitting results of Equation (4) from the main text to the experimental crystallization degree vs. time dependence during the isothermal crystallization of 3F5FPhH6 for crystallization temperatures T_{cr} from the 289-295 K range.

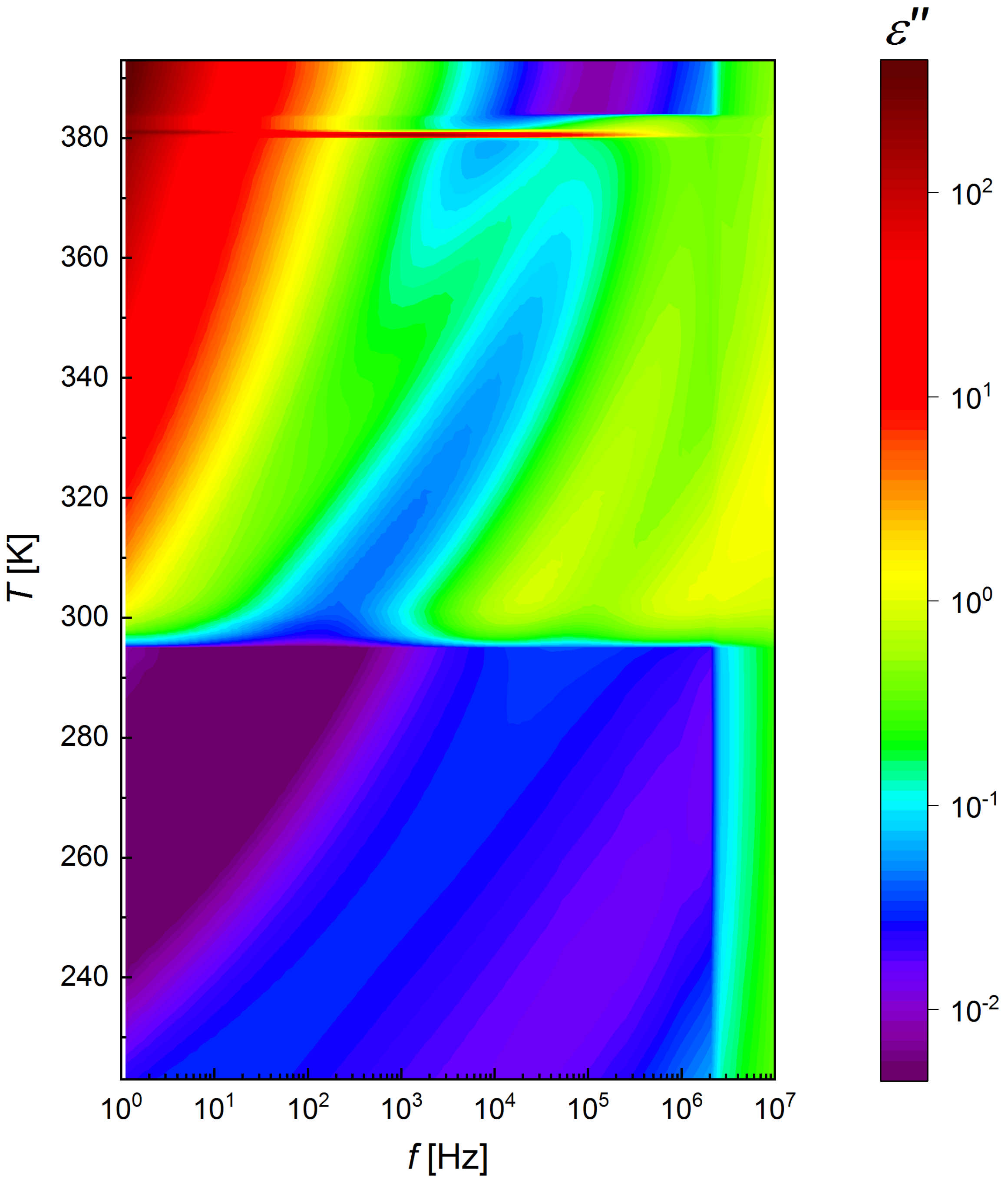

Figure S2. Dielectric absorption vs. temperature and frequency of 3F5FPhH6 on cooling.

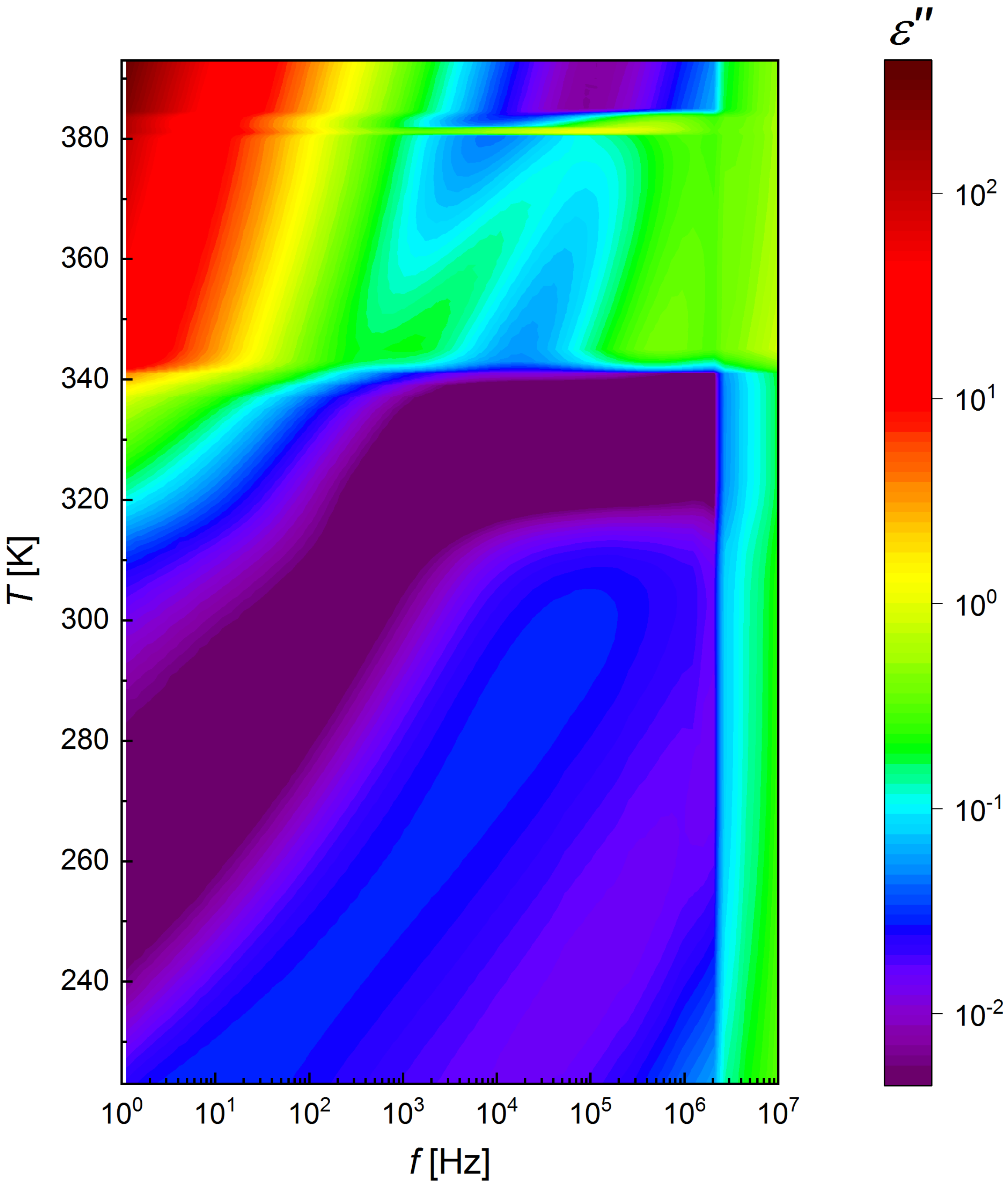

Figure S3. Dielectric absorption vs. temperature and frequency of 3F5FPhH6 on heating.

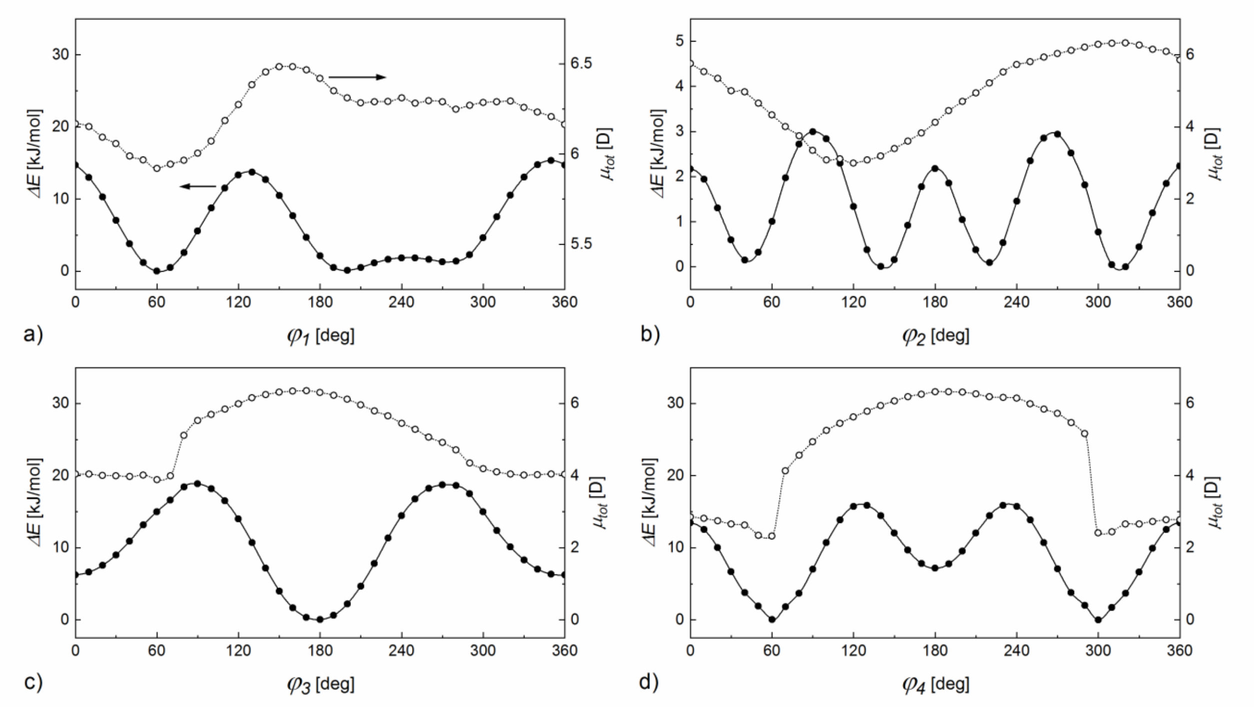

Figure S4. Conformational energy (solid symbols, left axis) and total dipole moment (open symbols, right axis) vs. torsional angle for selected intra-molecular rotations calculated for the isolated 3F5FPhH6 molecule with the semi-empirical PM7 method.

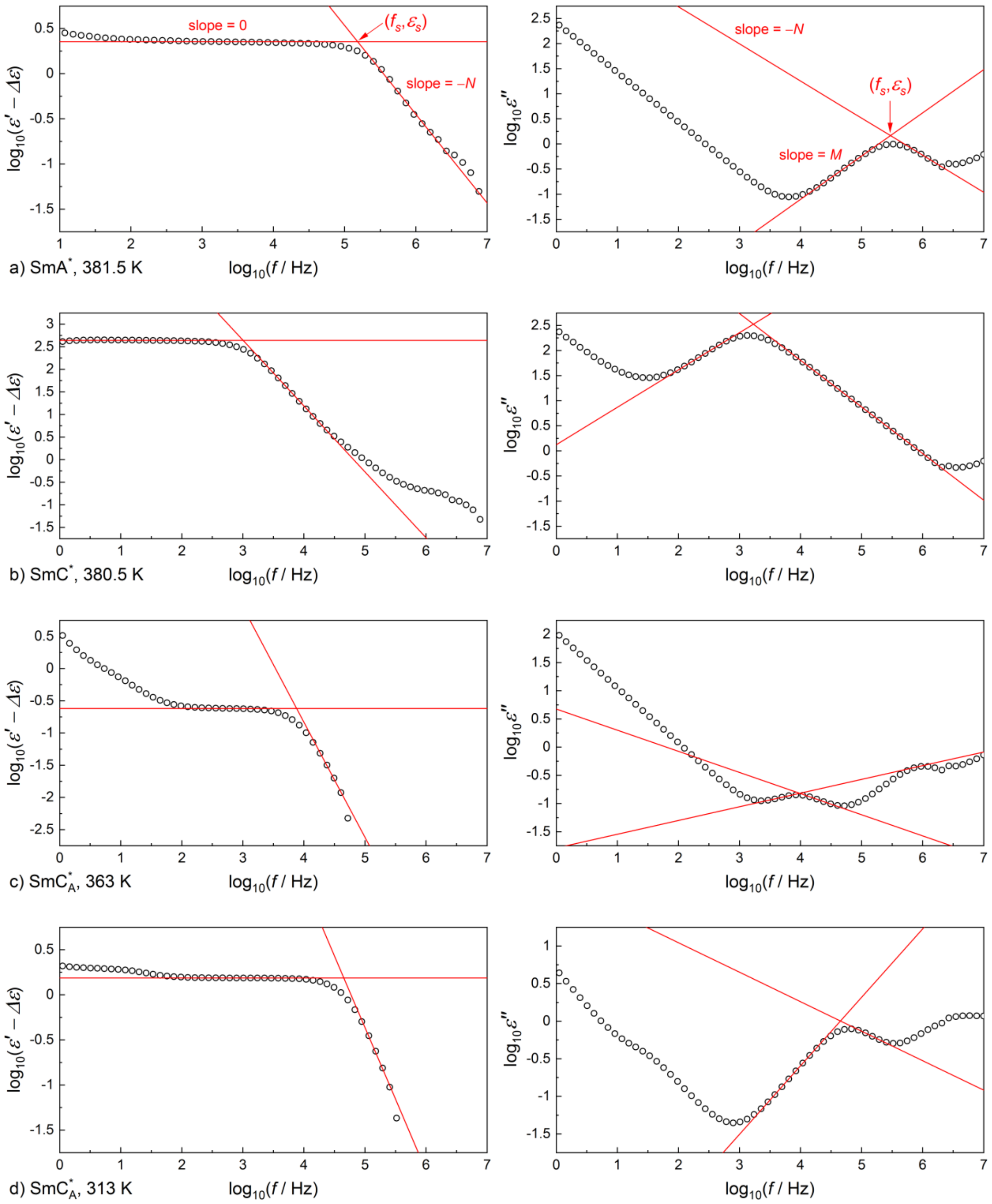

Figure S5. Determination of the M, N, f_s, ε_s parameters of Equation (6) for the relaxation processes: soft mode (a), Goldstone mode (b), P_L phason (c) and P_H phason (d) in the smectic phases of 3F5FPhH6.

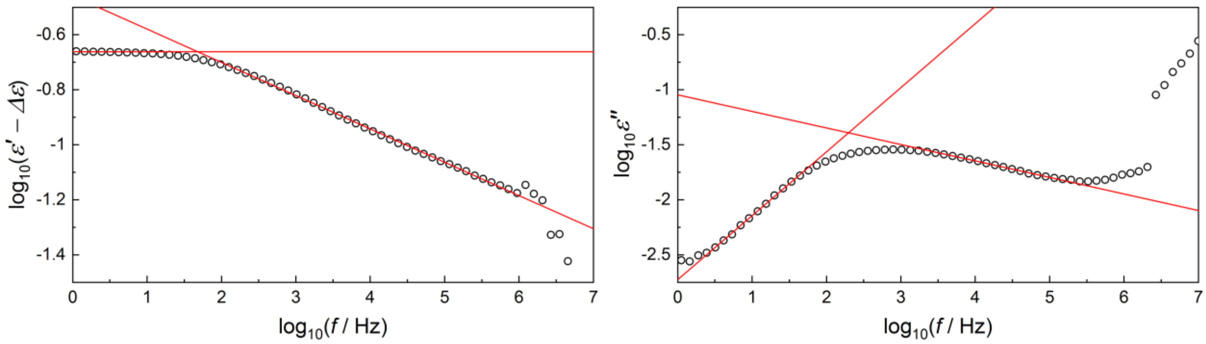

Figure S6. Determination of the M, N, f_s, ε_s parameters of Equation (6) for the cr-II process in the Cr2 phase of 3F5FPhH6.
